# Synthesis of Ergosterol Peroxide Conjugates as Mitochondria Targeting Probes for Enhanced Anticancer Activity

**DOI:** 10.3390/molecules24183307

**Published:** 2019-09-11

**Authors:** Ming Bu, Hongling Li, Haijun Wang, Jing Wang, Yu Lin, Yukun Ma

**Affiliations:** 1 College of Pharmacy, Qiqihar Medical University, Qiqihar 161006, China; LHL28@qmu.edu.cn (H.L.); wanghaijun@qmu.edu.cn (H.W.); qyyx305@qmu.edu.cn (J.W.); linyu7373@163.com (Y.L.); 2 Research Institute of Medicine & Pharmacy, Qiqihar Medical University, Qiqihar 161006, China; mayukun@qmu.edu.cn

**Keywords:** ergosterol peroxide, target probe, mitochondria, fluorescence imaging, antitumor activity

## Abstract

Inspired by the significant bioactivity of ergosterol peroxide, we designed and synthesized four fluorescent coumarin and ergosterol peroxide conjugates **8a**–**d** through the combination of ergosterol peroxide with 7-*N*,*N*-diethylamino coumarins fluorophore. The cytotoxicity of synthesized conjugates against three human cancer cells (HepG2, SK-Hep1, and MCF-7) was evaluated. The results of fluorescent imaging showed that the synthesized conjugates **8a**–**d** localized and enriched mainly in mitochondria, leading to significantly enhanced cytotoxicity over ergosterol peroxide. Furthermore, the results of biological functions of **8d** showed that it could suppress cell colony formation, invasion, and migration; induce G2/M phase arrest of HepG2 cells, and increase the intracellular ROS level.

## 1. Introduction

**Figure 1:**
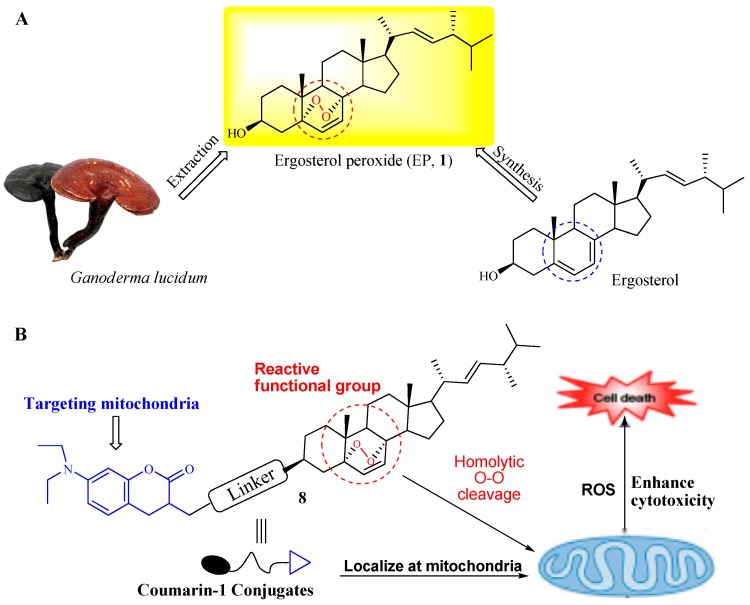
The chemical structures of compounds and design strategy. (**A**) The chemical structure and source of ergosterol peroxide (**1**). (**B**) The chemical structure of designed coumarin-**1** conjugates **8**, and the proposed mechanism of **8** for intracellular tracking and cancer-cell therapy.

Sterols are the important active ingredients of plants or fungal secondary metabolites. Steroidal compounds have drawn attention not only due to unusual and interesting chemical structures but also due to their widespread application as anti-inflammatory, diuretic, anabolic, and anticancer agents [1,2,3,4]. Sterol 5α,8α-endoperoxides are a kind of important active substances in drug discovery, which all have a unique 5α,8α-peroxy moiety (peroxidic bond or peroxide bridge) in the parent structure [5,6,7,8,9,10]. Ergosterol peroxide (5α,8α-epidioxiergosta-6,22-dien-3β-ol, EP, **1**) is a member of a class of fungal secondary metabolites of sterol endoperoxide derivatives and has been constantly discovered and extracted from different sources of medicinal fungi (Figure 1A) [11,12,13,14]. It has been reported that ergosterol peroxide can inhibit tumor cells growth by anti-angiogenesis or cytotoxicity [15,16,17,18,19].

In our previous study, we obtained pure ergosterol peroxide by chemical extraction and separation from *Ganoderma lucidum*. As the isolated amount of ergosterol peroxide from natural sources is not sufficient for an in-depth study on it, we got an effective chemical method to synthesize ergosterol peroxide (Figure 1). The synthesis of ergosterol peroxide was achieved by the treatment of natural ergosterol with oxygen in the presence of visible light and photosensitive catalysis [20,21,22]. Also, we have proved that ergosterol peroxide could inhibit forkhead-box O3 transcription factor (Foxo3a) functions by the inhibition of phosphorylate protein kinase (pAKT) to induce tumor cell death [23].

Natural ergosterol, which is lacking a peroxidic bond (O-O), has been proved with no significant activity against most of the cancer cells [24]. Hence, it is generally accepted that the peroxide bridge is a crucial functional group to the biological activities [25,26]. The hemolytic cleavage of the peroxide bridge in a reducing environment induces reactive oxygen species (ROS), which could be cytotoxic to cancer cells [27,28,29,30].

Selective delivery of drug probes to subcellular organelles in tumor cells has emerged as an effective and attractive strategy for cancer therapy. It exerts synchronous tumor targeting and molecular imaging of drug delivery. Therefore, various theranostic probes that target different subcellular organelles in cancer cells, including the endoplasmic reticulum, lysosomes, and mitochondria, have been continuously reported in recent years [31,32,33]. Among them, mitochondria represent the most attractive target for effective theranostic probe development due to their key role in controlling many essential functions in cancer cells. The specific motivation of inducing mitochondrial dysfunctions has been acknowledged as an effective method in cancer therapy [34,35,36,37]. 

Herein, we designed four fluorescent coumarin and ergosterol peroxide conjugates **8a**–**d** through the combination of ergosterol peroxide with different targeting coumarin-3-carboxamide analogs. We postulated that the mitochondria-targeting coumarin analogs could efficiently take ergosterol peroxide selective accumulated in mitochondria, in which subsequent production of ROS from the hemolytic cleavage of the endoperoxide bond in ergosterol peroxide would induce mitochondrial dysfunction and kill tumor cells (Figure 1B).

## 2. Results and Discussion

### 2.1. Chemistry

**Scheme 1:**
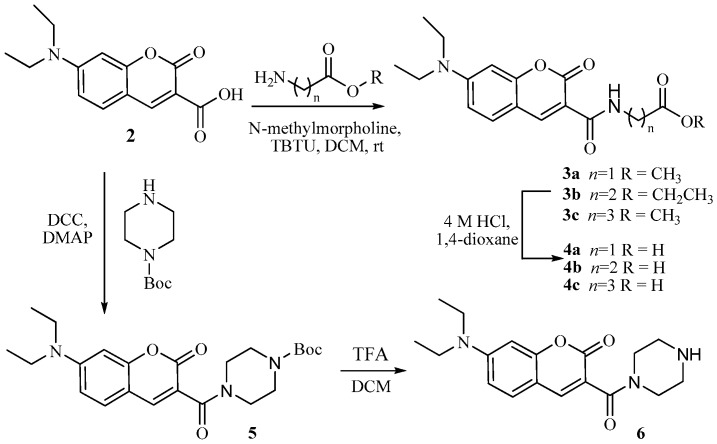
Synthesis of coumarin analogs **4a**–**c** and **6**.

First, using ergosterol (EG) as material, we achieved a chemical synthesis of ergosterol peroxide by the treatment of ergosterol in the presence of eosin Y, oxygen, and visible light (Scheme 1) [22].

There is a hydroxyl group at the C-3 position of ergosterol peroxide that is also a feasible functional site. We designed and synthesized four ergosterol peroxide-coumarin conjugates **8a**–**d** to evaluate the biological activities of various groups of linker at the C-3 position. The reason for the use of amino acids as spacers is that the amide bond is fairly stable, and the synthesis of compounds in which the amide bond is formed by coupling reagents is simple. Thus, three different amino acids (glycine, β-alanine, or γ-aminobutyric acid) were selected as the linker, to increase the space distance between ergosterol peroxide and coumarin fluorophore. 

**Scheme 2:**
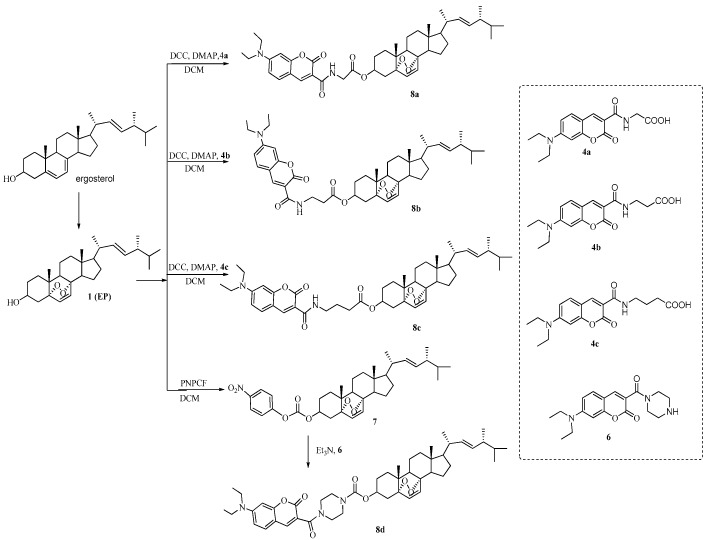
Synthesis of coumarin and ergosterol peroxide conjugates **8a**–**d**.

The synthetic routes of different coumarin fluorophore analogs are presented in Scheme 2. 2.7-(*N*,*N*-Diethylamino)coumarin-3-carboxylic acid (**2**) was generated according to known procedures [38]. Three different amino acid esters were introduced to the carboxyl group of compound **2** for amide bond formation by the same acylation reaction to give compounds **3a**–**c**. Then, the ester protecting a group of compounds **3a**–**c** could be hydrolyzed with an aqueous solution of hydrochloric acid to give three coumarin fluorophore carboxylic acid analogs **4a**–**c**. Besides, as a comparison, we also synthesized an ergosterol peroxide and fluorophore conjugate **6** with piperazine as the linker.

Finally, conjugates **8a**–**c** were obtained by introducing ergosterol peroxide directly to the carboxylic group of **4a**–**c**, using dicyclohexylcarbodi imide (DCC) as the coupling reagent (Scheme 2). Also, ergosterol peroxide was reacted with 4-nitrophenyl chloroformate using pyridine as the base in dichloromethane to get intermediate **7**. Then, **7** reacted with intermediate **6** to obtain the desired conjugate **8d**.

### 2.2. Optical Properties and Subcellular Localization

First, the optical properties of four ergosterol peroxide conjugates **8a**–**d** were investigated (Table 1). In general, all four conjugates possess typical optical properties [39]. Especially, probe **8d** with a maximum excitation wavelength (*λ*_ex_) of about 469.5 nm and an emission wavelength (*λ*_em_) of about 404 nm. The large Stokes shift (65.5 nm) of probe **8d** ensured ideal photophysical properties for living cells fluorescence imaging studies. Thus, probe **8d** was chosen for further subcellular localization study.

**Figure 2:**
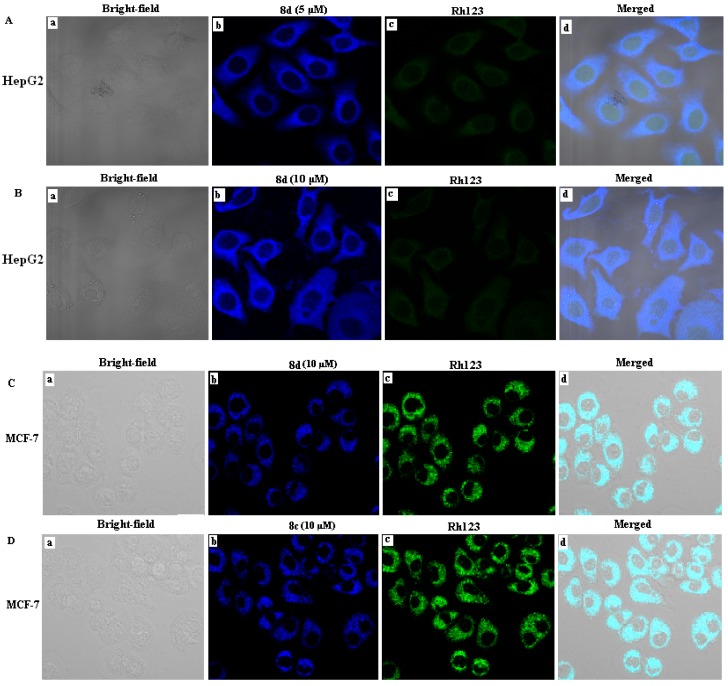
Confocal laser scanning microscopic images of cancer cells treated with **8c** or **8d** in the presence of Rh123 (0.5 μM, 20 min) in PBS buffer. (**A**) Fluorescence images of HepG2 cells stained with **8d** (5 μM). (**B**) Fluorescence images of HepG2 cells stained with **8d** (10 μM). (**C**) Fluorescence images of MCF-7 cells stained with **8d** (10 μM). (**D**) Fluorescence images of MCF-7 cells stained with **8c** (10 μM). (**a**) Cell images in bright-field. (**b**) Fluorescence images of cells stained with **8c** or **8d**. (**c**) Fluorescence images of cells stained with Rh123. (**d**) Merged images of (**b**) and (**c**). All images were acquired under a 20× objective lens.

The subcellular localization of probe **8d** in living HepG2 cells was carried with the green mitochondria-specific dye Rhodamine 123 (Rh123) as a comparison (*λ*_ex_ = 488 nm, *λ*_em_ = 515–530 nm) [40]. The intracellular microscopic fluoroscope imaging was captured by Carl Zeiss 710M confocal laser scanning microscopy. As shown in Figure 2A,B, HepG2 cells were universality stained by **8d** (5 μM and 10 μM) after 2 h of incubation. Bright blue fluorescence was captured successfully in HepG2 cells (*λ*_ex_ = 430–500 nm), which demonstrated both satisfactory permeability into the cell membrane and high cellular uptake of **8d**. What’s more, the extensive merged blue-green color fluorescence images illustrated that probe **8d** could strongly co-localize with specific dye Rh123 in mitochondria. Also, as shown in Figure 2C,D, MCF-7 cells were successfully stained by **8c** or **8d** (10 μM). The results suggested that the fluorescent coumarin-3-carboxamide could successfully deliver ergosterol peroxide to mitochondria.

### 2.3. Biological Evaluation

#### 2.3.1. Cytotoxic Activity

The newly synthesized conjugates **8a**–**d** and ergosterol peroxide were evaluated for their cytotoxicities against human hepatic carcinoma cells (HepG2, Sk-Hep2) and human breast cancer cells (MCF-7) by the MTT (3-(4,5-dimethylthiazol-2-yl)-2,5-diphenyltetrazolium bromide) assay. Cisplatin was chosen as a positive control drug. The results in Table 2 show that all conjugates have ideal cytotoxicity to these three tumor cell lines, with half maximal inhibitory concentration (IC_50_) values at the sub-micromolar levels. Among them, probes **8c** and **8d** were the most potent to the tested cancer cells. Probe **8c** with a longer linker (γ-aminobutyric acid) had around twofold improved potency against three tested cancer cells relative to that of **8a**. Besides, probe **8d** with a special pyridine moiety as linker exhibited significant cytotoxicity against human liver cancer cells (HepG2, SK-Hep1), with IC_50_ values of 6.60 μM for HepG2, which is nearly equivalent to **8c** for Sk-Hep1. Moreover, much weaker cytotoxicities were measured for either **1** or **2**, which suggested that the cytotoxicities of probes **8a**–**d** were associated with the synergistic effect of these two components. Overall, three probes **8b**, **8c,** and **8d** exhibited significant cytotoxicities against HepG2 cell lines, with IC_50_ values lower than 10 μM. To understand the mechanism of cancer cells death caused by treatment with coumarin-**1** conjugates, we chose probe **8d** for the series of biological functions experiments. 

#### 2.3.2. Effect of Probe 8d on the Cell Cycle Distribution

**Figure 3:**
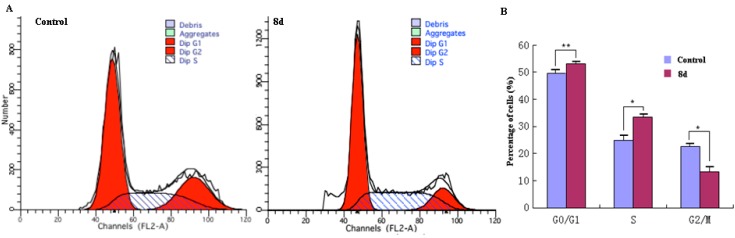
The effect of probe **8d** on the HepG2 cell cycle. (**A**) Typical distribution of cell cycles treated with or without **8d** (3 μM). (**B**) Statistical analysis of cells distributed in each phase of cell cycles. * *p* < 0.05, ** *p* < 0.01.

The effect of probe **8d** on tumor cell cycle distribution was carried by flow cytometry. We treated HepG2 cells with probe **8d** (3 μM) for 24 h. The low concentration of **8d** was used to avoid induction of HepG2 cell death. As shown in Figure 3A, in HepG2 cells, the cell phase distribution was 49.30 ± 1.03% vs. 53.12 ± 1.12% in G0/G1 phase, 25.09 ± 1.18% vs. 33.49 ± 1.05% in S phase, and 22.89 ± 1.17% vs. 12.31 ± 2.05% in G2/M phase before and after the effect of **8d**. The G0/G1 and S phases were increased significantly before and after the effect of **8d**, suggesting a chemical effect of **8d** on cell cycle progression. Also, the number of cells decreased in G2/M phases (Figure 3).

#### 2.3.3. Effect of Probe **8d** on the Cell Colony, Migration, and Invasion

**Figure 4:**
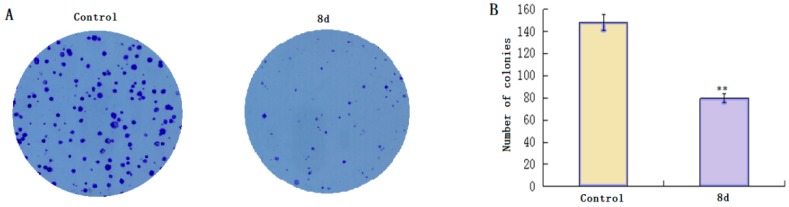
Compound **8d** inhibited cell colony formation. (**A**) Representative images of HepG2 cells colonies after treatment with **8d** (7 μM). The plates were stained with Coomassie Blue. (**B**) Data are presented as the mean ± SD of three experiments. ** *p* < 0.01.

The clonogenic assay is an effective method to evaluate the neoplastic transformation indirectly. Hence, we tested the effect of probe **8d** on HepG2 cells colony formation. The cells were treated with 7 μM probe **8d** for 20 days. The colony formation was observed under light microscopy. The colony numbers were 61.3 ± 3.1 vs. 9.1 ± 1.4 in HepG2 cells before and after the effect of **8d** (Figure 4). The results suggested that probe **8d** had a significant inhibitory activity to HepG2 cells growth.

**Figure 5:**
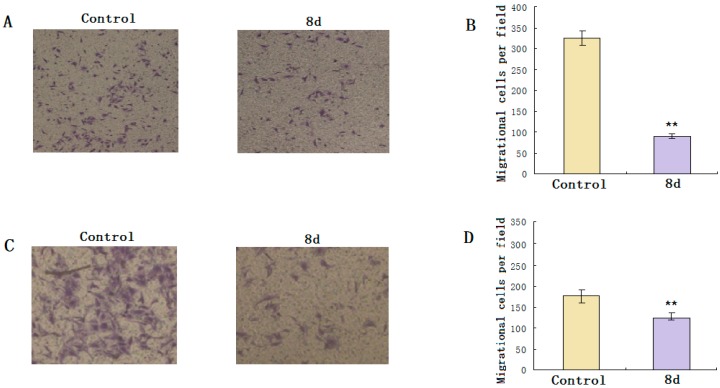
Compound **8d** suppressed HepG2 cell invasion and migration (Transwell). (**A**) Cell migration in HepG2 cells before and after the effect of **8d** (6 μM). (**B**) The migratory cell number is presented by bar diagram. (**C**) Cell invasion in HepG2 cells before and after the effect of **8d** (6 μM). (**D**) The number of cells invading to the lower chamber is presented by bar diagram. Data are presented as the mean ± SD of three experiments ** *p* < 0.01. All images were acquired under a 10× objective lens.

To determine if **8d** can prevent cancer cell invasion and migration, transwell assays were performed. Under the transwell assay, the number of migratory cells was 120.4 ± 4.2 vs. 63.2 ± 3.5 for HepG2 cells before and after the effect of **8d** (6 μM). The HepG2 cell number was reduced significantly after the effect of **8d**. The results suggested that **8d** suppressed HepG2 cancer cells migration (Figure 5A,B). The number of invasive cells was 107.1 ± 5.2 vs. 52.7 ± 4.6 for HepG2 cells before and after the effect of **8d** (6 μM). The HepG2 cell number was reduced significantly after the effect of 8**d**. The results suggested that **8d** suppressed HepG2 cancer cell migration and invasion (Figure 5C,D).

#### 2.3.4. Effect of **8d** on the Level of ROS

**Figure 6:**
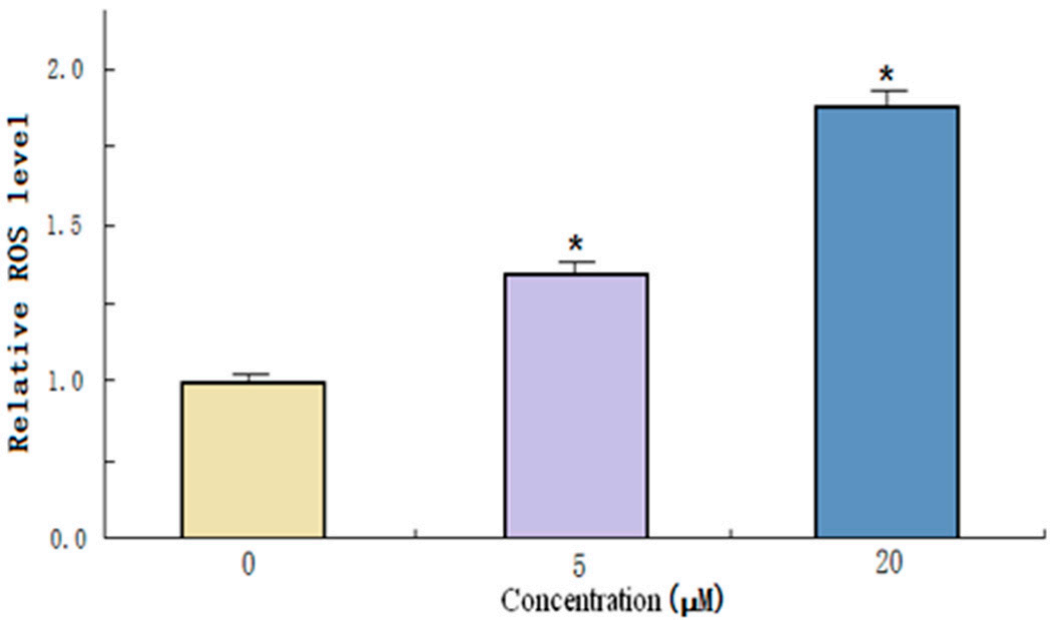
The intracellular ROS (reactive oxygen species) level of HepG2 cells was increased by treating with different concentrations of probe **8d**. Data are presented as the mean ± SD of three experiments. * *p* < 0.05.

To comprehend the mechanism of tumor cells death caused by treatment with probe **8d**, the level of intracellular ROS in HepG2 cells was monitored by using 5-(and-6)-chloromethyl-2’,7’-dichlorodihydrofluorescein diacetate acetyl ester (CM-H2DCFDA) as an indicator and detected by flow cytometry [41]. Figure 6 shows the effect of probe **8d** on ROS generation in HepG2 cells. A concentration-dependent increase in the ROS level was observed for HepG2 cell lines after incubation with probe **8d** (5 and 20 μM) for 24 h. The relative ROS level in HepG2 cancer cells, resulting from incubation with 20 μM probe **8d**, was about twofold higher than that of control cells without **8d**. This suggested that the intracellularly generated ROS was responsible for cell death.

## 3. Materials and Methods

### 3.1. Chemistry

Reagents were commercially available and used without purification. The ^1^H- and ^13^C-NMR spectra were recorded using the Bruker Avance DRX400 spectrometer (400 MHz and 100 MHz) in CDCl_3_ or DMSO-*d*_6_. Tetramethylsilane (TMS) was used as internal standards. Melting points were measured by an MP120 point apparatus. Mass spectra were measured in electrospray (ESI) mode on a mass spectrometer (Esquire 6000). Flash chromatography was performed using 400 mesh silica gel. ^1^H- and ^13^C-NMR spectra of new compounds are available online (See Supplementary Materials).

#### 3.1.1. Synthesis of Ergosterol Peroxide (1)

Ergosterol (150 mg), eosin (1 mg), and pyridine (20 mL) were added into a quartz tube. The mixture was kept in a water-cooled bath and vigorously stirred by the gas bubbling (O_2_), radiating with an iodine tungsten lamp (500 W, 220 V) for 0.5 h. After the reaction, the mixture was poured into ice-water (20 mL) and then extracted with 50 mL ethyl acetate twice. The ethyl acetate phase was washed with 50 mL saturated brine twice and then dried with anhydrous Na_2_SO_4_. The crude product was purified by chromatographic column (ethyl acetate/petroleumether = 1/5) to get pure ergosterol peroxide as white solid [22].

#### 3.1.2. Synthesis of 7-(Diethylamino)-2-Oxo-2*H*-Chromene-3-Carboxylic Acid (**2**) 

2,2-Dimethyl-1,3-dioxane-4,6-dione (1.5 g, 10.36 mmol), 4-(diethylamino)-2-hydroxy- benzaldehyde (2 g, 10.36 mmol), piperidine (0.08 g, 1.04 mmol), and acetic acid (two drops) in ethanol (20 mL) was stirred for 0.5 h at room temperature, and then the mixture was heated to reflux for 4 h. After the reaction, the mixture was poured into 50 mL of ice water. The orange precipitate was collected and washed with ethanol to give **2** the crystalline solid form (63%) [38].

#### 3.1.3. Synthesis of Amino-Acid Ester Derivatives

Under N_2_, dry methanol or ethanol (100 mL) was cooled down to −5 °C, and SOCl_2_ (21 mL, 0.3 mmol) was added dropwise. Glycine, β-alanine, or γ-aminobutyric acid (0.1 mmol) was added to this solution, and stirring was continued at room temperature for 3 h. The solvent was removed under reduced pressure to obtain the correspondent pure product as colorless crystals.

#### 3.1.4. Synthesis of 2-(7-(Diethylamino)-2-Oxo-2*H*-Chromene-3-Carboxamido Ester Derivatives **3a**–**c**

To a stirred solution of **2** (2.42 g, 9.27 mmol) in dry dichloromethane (DCM) (160 mL), *N*-methylmorpholine (4.08 mL, 37.1 mmol) and 2-(1H-Benzotriazole-1-yl)-1,1,3,3- tetramethyluronium tetrafluoroborate (TBTU) (4.46 g, 13.9 mmol) were added. After stirring for 20 min, methyl 2-aminoacetate chloride salt (ethyl 3-aminopropanoate or methyl 4-aminobutanoate) (13.9 mmol) was added. The reaction was continued at room temperature for approximately 1.5 h under argon atmosphere. The reaction was followed on TLC chromatography plates using CH_2_Cl_2_/CH_3_OH (5/1) as the eluent. The reaction mixture was washed with a saturated water solution of NaHSO_4_ (8 × 40 mL) and then dried with anhydrous Na_2_SO_4_. The crude product was purified by chromatographic column (10% MeOH in DCM) to give the desired products.

*Methyl 2-(7-(Diethylamino)-2-Oxo-2H-Chromene-3-Carboxamido)Acetate Ester* (**3a**): Yellow crystals (62%): mp 176–178 °C. ^1^H-NMR (CDCl_3_, 400 MHz) *δ* (ppm): 9.20 (1H, t, *J* = 5.60 Hz, -CONH-CH_2_-), 8.70 (1H, s, H^4^-Ar), 7.40 (1H, d, *J* = 8.40 Hz, H^5^-Ar), 6.65 (1H, dd, *J* = 2.60, 8.20 Hz, H^6^-Ar), 6.50 (1H, d, *J* = 3.60 Hz, H^8^-Ar), 4.24 (2H, d, *J* = 6.20 Hz, -CONH-CH_2_-), 3.78 (3H, s, -COO-CH_3_), 3.44 (4H, q, *J* = 7.10 Hz, 2 × -CH_2_CH_3_), 1.25 (6H, t, *J* = 7.10 Hz, 2 × -CH_2_CH_3_). ^13^C-NMR (CDCl_3_, 100 MHz) *δ* (ppm): 170.54, 163.91, 162.93, 158.14, 153.09, 148.70, 131.59, 110.40, 110.04, 108.67, 96.97, 52.621, 45.48, 41.93, 12.80. MS (ESI) *m/z*: [M + H]^+^ 333.1.

*Ethyl 3-(7-(Diethylamino)-2-Oxo-2H-Chromene-3-Carboxamido)Propanoate* (**3b**): Yellow crystals (65%): mp 129–133 °C. ^1^H-NMR (CDCl_3_, 400 MHz) *δ* (ppm): 9.09 (1H, t, *J* = 7.20 Hz, -CONH-CH_2_CH_2_-COO-), 8.71 (1H, s, H^4^-Ar), 7.44 (1H, d, *J* = 8.90 Hz, H^5^-Ar), 6.64 (1H, dd, *J* = 4.20, 7.70 Hz, H^6^-Ar), 6.51 (1H, d, *J* = 3.60 Hz, H^8^-Ar), 4.20 (2H, m, -COO-CH_2_CH_3_), 3.72–3.77 (2H, m, -CONH-CH_2_CH_2_-COO-), 3.48 (4H, q, *J* = 7.20 Hz, 2 × -CH_2_CH_3_), 2.67 (2H, m, -CH_2_CH_2_-COO-), 1.24–1.33 (9H, m, 2 × -CH_2_CH_3_, -COO-CH_2_CH_3_). ^13^C-NMR (CDCl_3_, 100 MHz) *δ* (ppm): 172.16, 163.48, 162.83, 157.95, 152.88, 148.28, 131.41, 110.46, 110.27, 108.60, 96.83, 60.97, 45.40, 35.59, 34.82, 30, 14.54, 12.78. MS (ESI) *m/z*: [M + H]^+^ 361.2.

*Methyl 4-(7-(Diethylamino)-2-Oxo-2H-Chromene-3-Carboxamido)Butanoate* (**3c**): Yellow crystals (70%): mp 150–152 °C. ^1^H-NMR (CDCl_3_, 400 MHz) *δ* (ppm): 8.82 (1H, t, *J* = 7.70 Hz, -CONH-CH_2_-), 8.70 (1H, s, H^4^-Ar), 7.43 (1H, d, *J* = 8.60 Hz, H^5^-Ar), 6.65 (1H, dd, *J* = 4.20, 7.60 Hz, H^6^-Ar), 6.49 (1H, d, *J* = 4.60 Hz, H^8^-Ar), 3.67 (3H, s, -COO-CH_3_), 3.42–3.52 (6H, m, 2 × -CH_2_CH_3_, -CONH-CH_2_CH_2_CH_2_-COO-), 2.42 (2H, t, *J* = 8.20 Hz, -CONH-CH_2_CH_2_CH_2_-COO-), 1.91–2.00 (2H, m, -CONH-CH_2_CH_2_CH_2_-COO-), 1.24 (6H, t, *J* = 7.40 Hz, 2 × -CH_2_CH_3_). ^13^C-NMR (CDCl_3_, 100 MHz) *δ* (ppm): 173.87, 163.61, 163.09, 157.99, 152.92, 148.41, 131.50, 110.61, 110.34, 108.73, 96.94, 51.97, 45.44, 39.22, 31.91, 25.38, 12.80. MS (ESI) *m/z*: [M + H]^+^ 361.2.

#### 3.1.5. Synthesis of 2-(7-(Diethylamino)-2-Oxo-*2H*-Chromene-3-Carboxamido)Carboxylic Acids **4a**–**c**

Four molar HCl (60 mL) was added dropwise into a stirred solution of **3a**–**c** (6 mmol) in 1,4-dioxane (120 mL). Stirring was continued at room temperature for 2 h. The reaction course was followed on TLC. The pH was adjusted to 3 with NaOH. The mixture was extracted with DCM (3 × 50 mL) and then dried with anhydrous Na_2_SO_4_. The crude product was purified by chromatographic column (DCM to 10% MeOH in DCM) to give the desired products **4a**–**c**.

*2-(7-(Diethylamino)-2-Oxo-2H-Chromene-3-Carboxamido)Acetic Acid* (**4a**): Yellow crystals (69%): mp 283–287 °C. ^1^H-NMR (CDCl_3_, 400 MHz) *δ* (ppm): 12.74 (1H, s, -COOH), 8.98 (1H, t, *J* = 5.80 Hz, -CONH-CH_2_-), 8.71 (1H, s, H^4^-Ar), 7.73 (1H, d, *J* = 8.00 Hz, H^5^-Ar), 6.84 (1H, m, H^6^-Ar), 6.67 (1H, d, *J* = 2.30 Hz, H^8^-Ar), 4.07 (2H, d, *J* = 8.20 Hz, -CONH-CH_2_-), 3.51 (4H, m, 2 × -CH_2_CH_3_), 1.18 (6H, t, *J* = 7.20 Hz, 2 × -CH_2_CH_3_). ^13^C-NMR (CDCl_3_, 100 MHz) *δ* (ppm): 172.95, 161.53, 161.41, 157.20, 152.33, 147.53, 131.48, 109.99, 109.45, 107.61, 95.84, 44.31, 43.53, 12.29. MS (ESI) *m/z*: [M + H]^+^ 317.1.

*3-(7-(Diethylamino)-2-Oxo-2H-Chromene-3-Carboxamido)Propanoic Acid* (**4b**): Yellow crystals (71%): mp 202–205 °C. ^1^H-NMR (DMSO-*d_6_*, 400 MHz) *δ* (ppm): 12.30 (1H, s, -COOH), 8.82 (1H, d, *J* = 8.20 Hz, -CONH-CH_2_-), 8.65 (1H, s, H^4^-Ar), 7.65–7.71 (1H, m, H^5^-Ar), 6.65-6.83 (1H, m, H^6^-Ar), 6.60 (1H, s, H^8^-Ar), 3.41–3.61 (6H, m, 2 × -CH_2_CH_3_, -CONH-CH_2_-), 2.45 (2H, m, -CONH-CH_2_CH_2_-COOH), 1.14 (6H, t, *J* = 7.80 Hz, 2 × -CH_2_CH_3_). ^13^C-NMR (DMSO-*d_6_*, 100 MHz) *δ* (ppm): 174.02, 163.02, 162.51, 158.06, 153.26, 148.58, 132.40, 110.92, 110.08, 108.52, 96.69, 45.21, 35.76, 34.80, 13.16. MS (ESI) *m/z*: [M + H]^+^ 333.1.

*4-(7-(Diethylamino)-2-Oxo-2H-Chromene-3-Carboxamido)Butanoic Acid* (**4c**): Yellow crystals (70%): mp 198–200 °C. ^1^H-NMR (DMSO-*d*_6_, 400 MHz) *δ* (ppm): 12.06 (1H, s, -COOH), 8.62–8.66 (2H, m, -CONH-CH_2_-, H^4^-Ar), 7.67 (1H, d, *J* = 8.00 Hz, H^5^-Ar), 6.80 (1H, dd, *J* = 3.40, 7.20 Hz, H^6^-Ar), 6.61 (1H, d, *J* = 4.60 Hz, H^8^-Ar), 3.48 (4H, m, 2 × -CH_2_CH_3_), 3.27–3.33 (2H, m, -CONH-CH_2_CH_2_CH_2_-), 2.26 (2H, d, *J* = 7.80 Hz, -CONH-CH_2_CH_2_CH_2_-COOH), 1.69–1.79 (2H, m, -CONH-CH_2_CH_2_CH_2_-COOH), 1.14 (6H, d, *J* = 7.40 Hz, 2 × -CH_2_CH_3_). ^13^C-NMR (DMSO-*d*_6_, 100 MHz) *δ* (ppm): 175.02, 163.11, 162.59, 158.04, 153.24, 148.45, 132.36, 110.93, 110.40, 108.53, 96.72, 45.20, 32.11, 25.65, 13.17. MS (ESI) *m/z*: [M − H]^+^ 345.1.

#### 3.1.6. Synthesis of Intermediate **6**

To a DCM solution (20 mL) of coumarin acid **2** (992.8 mg, 3.8 mmol) and mono-*t*-Boc-piperazine (1061.3 mg, 5.7 mmol), DCC (1030.0 mg, 5.0 mmol) and DMAP (6.1 mg, 0.05 mmol) were added at room temperature [41]. The solution was stirred at room temperature overnight, and then the solvent was removed under reduced pressure. The crude product was purified by chromatographic column (DCM) to give **5** as yellow solid powder (87%). Compound **6** was obtained by treatment of **5** with trifluoroacetic acid: DCM (1:1) solution. ^1^H-NMR (400 MHz, DMSO-*d*_6_) *δ* (ppm): 8.89 (1H, s, NH), 8.03 (1H, s, H^4^-Ar), 7.51 (1H, d, *J* = 8.80 Hz, H^5^-Ar), 6.77 (1H, dd, *J* = 8.00, 3.2 Hz, H^6^-Ar), 6.55 (1H, d, *J* = 2.2 Hz, H^8^-Ar), 3.67 (4H, m, 2 × -CH_2_CH_3_), 3.52 (4H, m, 2 × -CH_2_CH_2_NH-), 3.20 (4H, m, 2 × -CH_2_CH_2_NH-), 1.13 (6H, t, *J* = 7.2 Hz, 2 × -CH_2_CH_3_,). MS (ESI) *m/z*: [M + H]^+^ 320.2.

#### 3.1.7. Synthesis of Intermediate **7**

To a stirred solution of ergosterol peroxide (200 mg, 0.56 mmol) and 4-nitrophenyl chloroformate (258 mg, 1.28 mmol) in DCM (5 mL) under N_2_ gas, pyridine (100 mg, 1.28 mmol) was added, and stirring continued at room temperature for 3 h. The reaction course was followed on TLC. Then, DCM (20 mL) and H_2_O (20 mL) were added into the mixture. The DCM phase was combined, washed with brine, and then dried with anhydrous Na_2_SO_4_. The solution was concentrated to give yellow residue **7** for the next step. 

#### 3.1.8. Synthesis of Novel Conjugates **8a**–**d**

General synthesis procedure of conjugates **8a**–**d**: Coumarin-3-carboxylic acid derivatives **4a** (**4b**, **4c**, or **7**) (0.20 mmol), DCC (40 mg, 0.20 mmol), and DMAP (10%) were added into the solution of ergosterol peroxide (68 mg, 0.13 mmol) in DCM (20 mL). The mixture was stirred continuously at room temperature for 24~36 h. The filtrate was collected and concentrated, and the crude product was purified by chromatographic column to give target probes **8a**–**d**.

*2-[7-(Diethylamino)-2-Oxo-2H-Chromene-3-Carboxamido]*-*Glycine-3-(Ergosterol Peroxide) Probe***8a**: Yellow powder (52%). ^1^H-NMR (400 MHz, CDCl_3_) *δ* (ppm): 9.20 (1H, t, *J* = 5.4 Hz, -CONH-CH_2_-), 8.71 (1H, s, H^4^-Ar), 7.42 (1H, dd, *J* = 8.8, 4.2 Hz, H^5^-Ar), 6.71–6.56 (1H, m, H-6), 6.50 (2H, m, H^6^-Ar, H^8^-Ar), 6.25 (1H, d, *J* = 8.7 Hz, H-7), 5.25–5.14 (2H, m, H-22, H-23), 5.11 (1H, m, -OH), 4.20 (2H, m, NHCH_2_CO), 3.46 (4H, dd, *J* = 8.2, 3.6 Hz, 2 × -CH_2_CH_3_), 2.22–2.16 (1H, m), 2.11–2.06 (1H, m), 2.02 (2H, dd, *J* = 9.8, 3.3 Hz), 1.98–1.90 (2H, m), 1.85 (1H, dd, *J* = 12.9, 6.8 Hz), 1.72 (1H, m), 1.65 (4H, m), 1.58 (1H, m), 1.50 (2H, dd, *J* = 10.7, 7.2 Hz), 1.43 (1H, m), 1.34 (2H, m), 1.28-1.24 (9H, m), 1.11 (1H, m), 1.01 (3H, d, *J* = 6.6 Hz, -CH_3_), 0.90 (6H, m, 2 × -CH_2_CH_3_), 0.82 (9H, dd, *J* = 8.3, 4.5 Hz, 3 × -CH_3_). ^13^C-NMR (100 MHz, CDCl_3_) *δ* (ppm): 168.6, 163.4, 162.6, 157.8, 152.7, 148.3, 136.2, 135.1, 132.3, 131.2, 130.8, 109.9, 109.8, 108.3, 96.7, 81.8, 79.4, 70.75, 56.2, 51.6, 51.0, 45.1, 44.6, 45.1, 44.5, 42.8, 39.7, 36.9, 34.2, 33.1, 29.7, 28.6, 26.2, 23.4, 20.9, 20.6, 19.9, 19.6, 18.1, 17.6, 12.9,12.4. MS (ESI) *m/z*: 727.4 [M + H]^+^.

*2-[7-(Diethylamino)-2-Oxo-2H-Chromene-3-Carboxamido]*-*Alanine-3-(Ergosterol Peroxide) Probe***8b**: Yellow powder (45%). ^1^H-NMR (400 MHz, CDCl_3_) *δ* (ppm): 9.06 (1H, t, *J* = 5.0 Hz, -CONH-CH_2_-), 8.68 (1H, s, H^4^-Ar), 7.42 (1H, d, *J* = 8.2 Hz, H^5^-Ar), 6.65 (1H, m, H-6), 6.50 (2H, t, *J* = 3.6 Hz, H^6^-Ar, H^8^-Ar), 6.25 (1H, d, *J* = 8.4 Hz, H-7), 5.21–5.15 (2H, m, H-22, H-23), 5.05 (1H, m, -OH), 3.72 (2H, m, -NHCH_2_CH_2_CO-), 3.46 (4H, dd, *J* = 8.0, 3.6 Hz, 2 × -CH_2_CH_3_), 2.62 (2H, t, *J* = 8.0, 3.8 Hz, -NHCH_2_CH_2_CO-), 2.11 (1H, m), 2.01 (1H, m), 1.99–1.94 (4H, m), 1.71–1.60 (4H, m), 1.50 (2H, m), 1.43 (2H, m), 1.34 (2H, m), 1.28–1.24 (9H, m), 1.11 (1H, m), 1.01 (3H, d, *J* = 6.6 Hz, -CH_3_), 0.91 (6H, m, 2 × -CH_2_CH_3_), 0.81 (9H, dd, *J* = 8.2, 4.4 Hz, 3 × -CH_3_). ^13^C-NMR (100 MHz, CDCl_3_) *δ* (ppm): 170.8, 163.2, 162.5, 157.7, 152.5, 148.1, 135.2, 134.1, 132.3, 131.1, 130.8, 110.2, 109.9, 108.3, 96.6, 81.8, 79.3, 69.9, 56.2, 51.6, 51.0, 45.1, 44.6, 42.7, 39.7, 39.3, 36.9, 34.7, 33.1, 33.0, 28.6, 25.6, 25.0, 20.9, 19.9, 19.6, 18.1, 17.6, 12.9, 12.4. MS (ESI) *m/z*: 741.4 [M + H]^+^.

*2-[7-(Diethylamino)-2-Oxo-2H-Chromene-3-Carboxamido]*-*Threonine-3-(Ergosterol Peroxide) Probe***8c**: Yellow powder (52%). ^1^H-NMR (400 MHz, CDCl_3_) *δ* (ppm): 8.84 (1H, s, -CONH-CH_2_-), 8.69 (1H, s, H^4^-Ar), 7.42 (1H, d, *J* = 9.0 Hz, H^5^-Ar), 6.65 (1H, dd, *J* = 9.0, 2.5 Hz, H-6), 6.50 (2H, dd, *J* = 5.4, 3.0 Hz, H^6^-Ar, H^8^-Ar), 6.23 (1H, d, *J* = 8.5 Hz, H-7), 5.18 (2H, qd, *J* = 15.3, 7.7 Hz, H-22, H-23), 5.05–4.94 (1H, m, -OH), 4.12 (6H, q, *J* = 7.1 Hz, -NHCH_2_CH_2_CH_2_CO-), 3.54–3.40 (4H, m, 2 × -CH_2_CH_3_), 2.36 (2H, d, *J* = 7.7 Hz), 2.05 (3H, m), 1.95–1.92 (4H, m), 1.72–1.32 (16H, m), 1.30-1.22 (6H, m, 2 × -CH_3_), 1.14–1.08 (4H, m), 0.92–0.88 (6H, m, 2 × -CH_2_CH_3_), 0.86–0.81 (6H, m, 2 × -CH_3_). ^13^C-NMR (100 MHz, CDCl_3_) *δ* (ppm): 173.6, 164.7, 163.8, 157.6, 157.0, 153.0, 148.1, 135.2, 135.1, 132.3, 131.1, 130.8, 109.9 108.5, 96.6, 81.7, 79.4, 69.5, 56.2, 51.6, 51.0, 49.1, 45.1, 44.5, 42.8, 39.7, 39.3, 36.7, 33.9, 33.1, 32.1, 29.7, 26.4, 25.6, 24.9, 23.4, 22.8, 20.9, 19.9, 19.6, 18.1, 17.6, 12.9, 12.4. MS (ESI) *m/z*: 755.4 [M + H]^+^.

*2-[7-(Diethylamino)-2-Oxo-2H-Chromene-3-Carboxamido]-Piperazine-3-(Ergosterol Peroxide) Probe***8d**: Yellow powder (49%). ^1^H-NMR (400 MHz, CDCl_3_) *δ* (ppm): 7.88 (1H, s, H^4^-Ar), 7.31 (1H, d, *J* = 8.80 Hz, H^5^-Ar), 6.60 (1H, dd, *J* = 9.00, 2.60 Hz, H-6), 6.48 (2H, dd, *J* = 12.0, 5.20 Hz, H^6^-Ar, H^8^-Ar), 6.23 (1H, d, *J* = 8.6 Hz, H-7), 5.19 (2H, *m*, H-22, H-23), 4.98–4.86 (1H, m, -OH), 3.71 (2H, s, -CH_2_CH_3_), 3.54 (4H, s, -NCH_2_CH_2_N-), 3.45 (4H, q, *J* = 7.20 Hz, -NCH_2_CH_2_N-), 3.37 (2H, s, -CH_2_CH_3_), 2.18 (1H, d, *J* = 4.60 Hz), 2.02 (4H, m), 1.85 (1H, d, *J* = 7.00 Hz), 1.76-1.69 (1H, m), 1.63 (4H, m), 1.59 (2H, dd, *J* = 13.0, 5.8 Hz), 1.52 (2H, t, *J* = 6.2 Hz), 1.39–1.35 (1H, m), 1.25 (4H, d, *J* = 5.20 Hz), 1.23 (6H, m, 2 × -CH_3_), 1.01 (3H, m, -CH_3_), 0.90 (6H, m, 2 × -CH_2_CH_3_), 0.81 (9H, m, 3 × -CH_3_). ^13^C-NMR (100 MHz, CDCl_3_) *δ* (ppm): 165.2, 159.2, 157.3, 154.6, 151.8, 135.1, 135.1, 132.4, 130.9, 130.0, 115.7, 109.4, 107.8, 97.0, 81.9, 79.4, 70.9, 56.3, 51.7, 51.1, 50.0, 44.6, 42.8, 39.7, 39.4, 37.0, 34.4, 33.5, 33.1, 28.7, 23.3, 21.0, 19.8, 19.6, 18.0, 17.6, 12.9, 12.4. MS (ESI) *m/z*: 784.5 [M + H]^+^

### 3.2. Biological Evaluation

#### 3.2.1. Cell Culture

Cancer cell lines (HepG2, SK-Hep1, and MCF-7) were obtained from the College of Pharmacy, Qiqihar Medical University. Cells were cultured in DMEM medium (HepG2 and SK-Hep1) or RPMI 1640 medium (MCF-7) supplemented with 10% FBS and 1% penicillin/streptomycin and maintained in an incubator with a humidified atmosphere of 5% CO_2_ at 37 °C [23].

#### 3.2.2. MTT Assay

The effects of compounds on cell viabilities of HepG2, SK-Hep1, and MCF-7 cells were evaluated by an MTT assay. Exponentially growing cells were seeded at a density of 2 × 10^4^ cells/mL into 96-well plates and cultured for 24 h. The tested samples at gradient concentrations (0.5, 1, 10, 20, 50, and 100 μM) were solubilized in DMSO. Then, the cells were treated with samples for 48 h and 10 μL of MTT (KeyGEN Biotech, Nanjing, China) was added for an extra 2 h. Control cells were treated with vehicle alone. The formazan dye product was measured on a Spectra Max 340 microplate reader at 490 nm (Thermo Multiskan MK3, Thermo Electron Corporation, Waltham, MA, USA). The IC_50_ values of all compounds were calculated using SPSS 17.0 software (SPSS, Chicago, IL, USA).

#### 3.2.3. Living Cell Staining for Subcellular Localization

HepG2 cells were incubated on glass cover-slips in 6-well plate (4 × 10^5^ cells/2 mL of medium for per well). After removing the medium, the cells were incubated with probe **8d** (5 µM and 10 µM) (in 2 mL of HBSS buffer per well) for 2 h, and then Rh123 (100 nM) was added in the last 30 min. After removing of HBSS buffer and three times of wash with PBS, the cells were fixed with 4% paraformaldehyde for 10 minutes and washed with PBS for three times. At last, the cover-slips were sealed strictly with nail polish for imaging.

#### 3.2.4. Confocal Microscopic Imaging

Confocal images were obtained with confocal microscopy (LSM 510, Carl Zeiss, Oberkochen, Germany), original magnification 100×. The excitation at 579 nm was used for Rh123 dye. For probe **8d**, the excitation laser was 404 nm, and the images were collected at the emission of 470 nm. The colocalization analysis of probe **8d** and Rh123 on images were processed by LSM 510 software (Carl Zeiss, Oberkochen, Germany).

#### 3.2.5. Cell Cycle Analysis

The HepG2 cells were cultivated at a concentration of 5 × 10^5^ cells/mL into 6-well plates with probe **8d** (3 µM) for 24 h. The cells were washed in PBS and centrifuged for 5 min at 350 g (Eppendorf 5804R, Hamburg, Germany) The collected pellets were incubated at a concentration of 10^6^ cells/mL with propidium iodide (PI, BD Pharmingen, BD Biosciences, San Jose, CA, USA) staining solution (50 µg/mL in PBS) for 30 min. The cells cycle was analyzed with flow cytometry (BD Biosciences).

#### 3.2.6. Colony, Invasion, And Migration Analysis

Colony formation: HepG2 cells (500 cells/well) were mixed with low melting agarose gel (0.25%). Then, probe **8d** (7 µM) was added into 6-well plates and maintained in 5% CO_2_ incubator for two weeks. The colonies of cells were counted and stained, and then the photograph was analyzed under an optics microscope. Migration: HepG2 cells (3 × 10^4^) were mixed in RPMI 1640 media (200 µL) and added to the upper chamber of transwell apparatus (8 µm pore size, COSTAR transwell, Corning, NY, USA). The cells were treated with **8d** (7 μM) for 20 h. Cells that migrated to the lower chamber were stained using crystal violet, and then the number of cells in three different high-power fields (×100) was counted. Invasion: the upper chamber of transwell was coated using a layer of the extracellular matrix. HepG2 cells (1 × 10^5^) were added to the upper chamber and treated with **8d** (7 μM) for 24 h. Then, the cells were stained and counted that invaded to the lower chamber in the same way as migration.

#### 3.2.7. Measurement of Intracellular ROS in HepG2 Cells

The production of ROS was measured using CM-H_2_DCFDA (Sigma-Aldrich, Milan, Italy). Briefly, HepG2 cells (5 × 10^5^) were seeded in 6 cm dishes and exposed to compound **8d** for 24 h at serial concentrations (0, 5, and 20 µM), after which the medium was removed, and washed with PBS, followed by incubation with 10 μM CM-H_2_DCFDA for 30 min at 37 °C in serum-free medium. Then, the incubation medium containing CM-H_2_DCFDA was removed, washed with PBS, and the cells were trypsinized and resuspended in PBS buffer. The quantity of ROS was monitored by flow cytometer (BD Biosciences), with dual channes fluorescence (DCF) luorescence detection channel.

## 4. Conclusions

In summary, we have successfully designed and prepared four mitochondria-targeting theranostic probes **8a**–**d** by direct conjugation of a coumarin-3-carboxamide fluorophore with ergosterol peroxide **1**. The cytotoxicities of synthesized conjugates against three human tumor cell lines (HepG2, SK-Hep1, and MCF-7) were investigated. The results of fluorescent imaging showed that the synthesized conjugates **8a**–**d** localized and enriched mainly in mitochondria, leading to significantly enhanced cytotoxicity over ergosterol peroxide. The biological functions of probe **8d** showed that it inhibited cell colony formation, invasion, and migration; induced G2/M phase arrest of HepG2 cells, and increased the intracellular ROS level. The present work provides new information to the potential of ergosterol peroxide as a drug lead compound for the versatile interest of research on steroidal anticancer agents. Besides, the strategy of natural products to mitochondria with enhanced cytotoxicity is in contrast to the well-established methods of using drug-mediated fluorophore delivery and may represent a feasible method for the design of theranostic probes.

## Figures and Tables

**Table 1:** Photophysical data for probes **8a**–**d** and **2** ^[a]^.

Entry	Probe	*λ*_ex_ (nm) ^[b]^	*λ*_em_ (nm) ^[c]^	Stokes Shift (nm)
1	**8a**	479	422.5	56.5
2	**8b**	475	422	53
3	**8c**	476	421.5	54.5
4	**8d**	469.5	404	65.5
5	**2**	470.5	406	64.5

^[a]^ Determined in DMSO (1 μM in DMSO). ^[b]^ Excitation maxima. ^[c]^ Emission maxima.

**Table 2:** Cytotoxicities of compounds against three cancer cell lines.

Comp.	IC_50_ (μM) ^[a]^		
	HepG2	SK-Hep1	MCF-7
**8a**	12.34 ± 0.49	10.43 ± 0.88	14.80 ± 0.28
**8b**	9.46 ± 0.35	11.70 ± 0.28	13.70 ± 0.28
**8c**	6.74 ± 0.32	5.92 ± 0.53	7.45 ± 0.50
**8d**	6.60 ± 0.16	8.10 ± 0.28	10.62 ± 0.10
**EP**(**1**)	23.15 ± 0.18	19.25 ± 0.60	26.06 ± 0.46
**2**	>100	>100	>100
Cisplatin	0.65 ± 0.05	2.42 ± 0.06	6.64 ± 0.16

^[a]^ Data represent the mean ± SD values of three independent determinations.

## Supplementary Materials

The following are available online at https://www.mdpi.com/1420-3049/24/18/3307/s1, Supplementary materials for ^1^H- and ^13^C-NMR spectra of new compounds are available online.

## Abbreviations

HepG2, SK-Hep1	Human liver carcinoma cell line
MCF-7	human breast adenocarcinoma cell line
DCC	dicyclohexylcarbodi imide
HOBT	1-hydroxylbenzotriazole
DMAP	4-dimethylaminopyridine
EDCI	1-ethyl-3-(3-dimethylaminopropyl)carbodiimide
PNPFC	4-nitrophenyl chloroformate
CM-H2DCFDA	5-(and-6)-chlorometry-2’,7’-dichlorodihydro-fluorescein diacetate acetyl ester
TBTU	2-(1H-Benzotriazole-1- yl)-1,1,3,3-tetramethyluronium tetrafluoroborate
DIPEA	N,N-diisopropylethylamine
