# Supplementary material for: Synthesis of Ergosterol Peroxide Conjugates as Mitochondria Targeting Probes for Enhanced Anticancer Activity
Source: Molecules. 2019 Sep 11;24(18):3307. doi: 10.3390/molecules24183307 (PMC6766909; doi:10.3390/molecules24183307)

molecules

MDPI

Molecules **2019**

Supplementary material

Synthesis of Ergosterol Peroxide conjugates as Mitochondria Targeting Probes for Enhanced Anticancer Activity

Ming Bu[1,*], Hongling Li[1], Haijun Wang[1], Jing Wang[1], Yu Lin[1] and Yukun Ma[2]

[1] College of Pharmacy, Qiqihar Medical University, Qiqihar 161006, China;

[2] Research Institute of Medicine & Pharmacy, Qiqihar Medical University, Qiqihar 161006, China.

* Correspondence: buming@qmu.edu.cn (M.B.); Tel.: +86-0452-2663-881

Ergosterol peroxide (EP, 1)

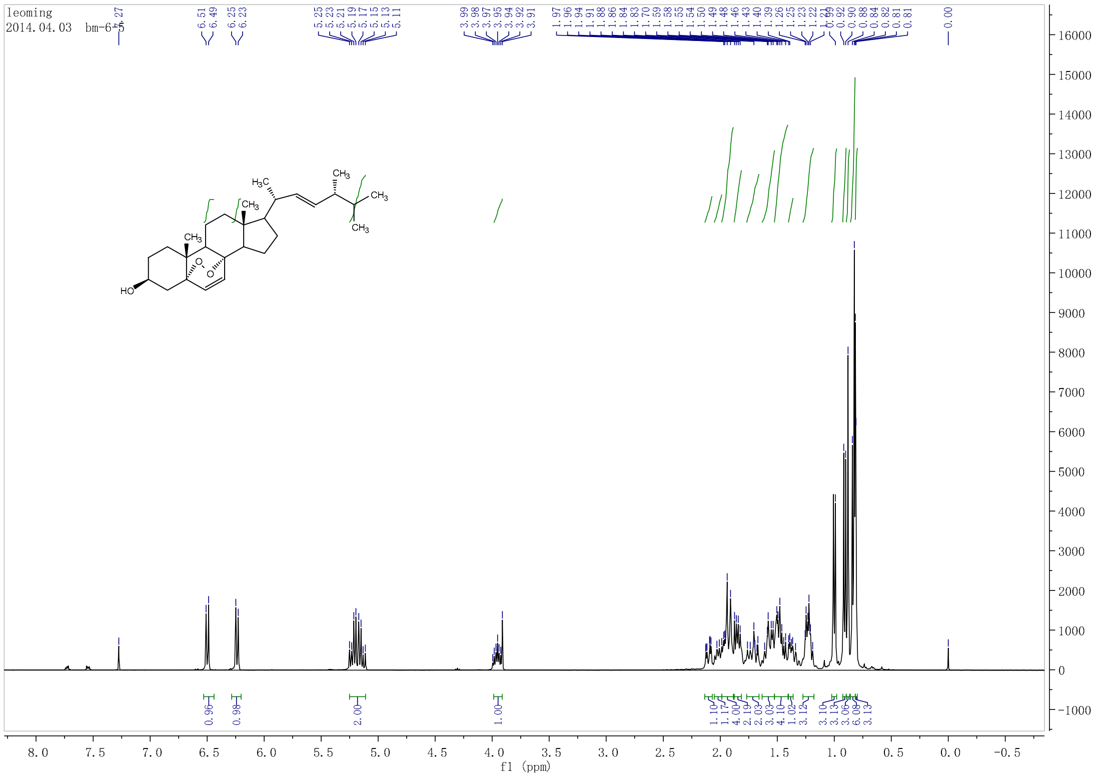

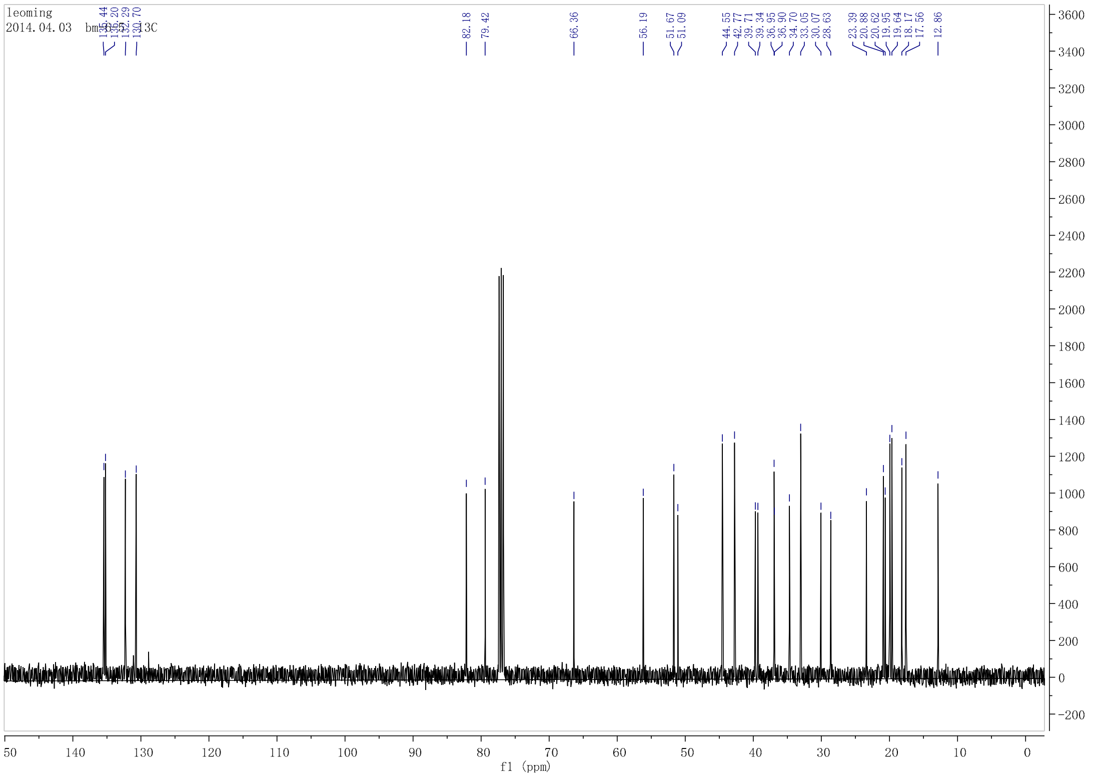

^{1}H NMR and ^{13}C NMR spectrums of **2**

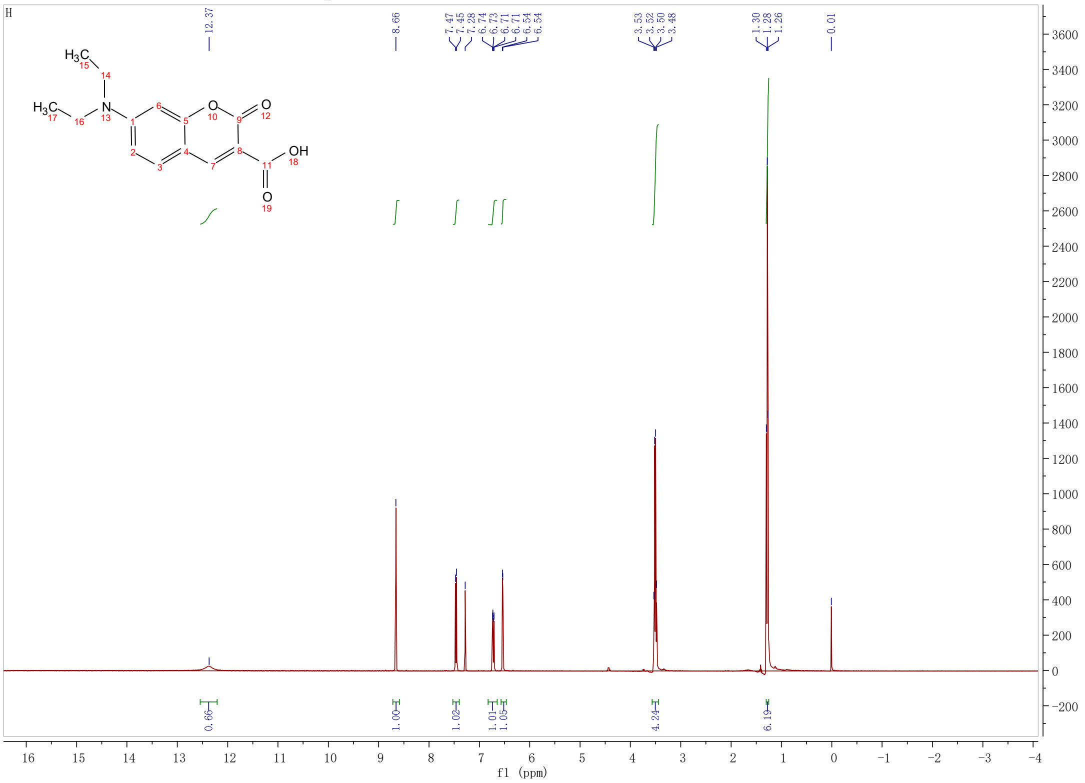

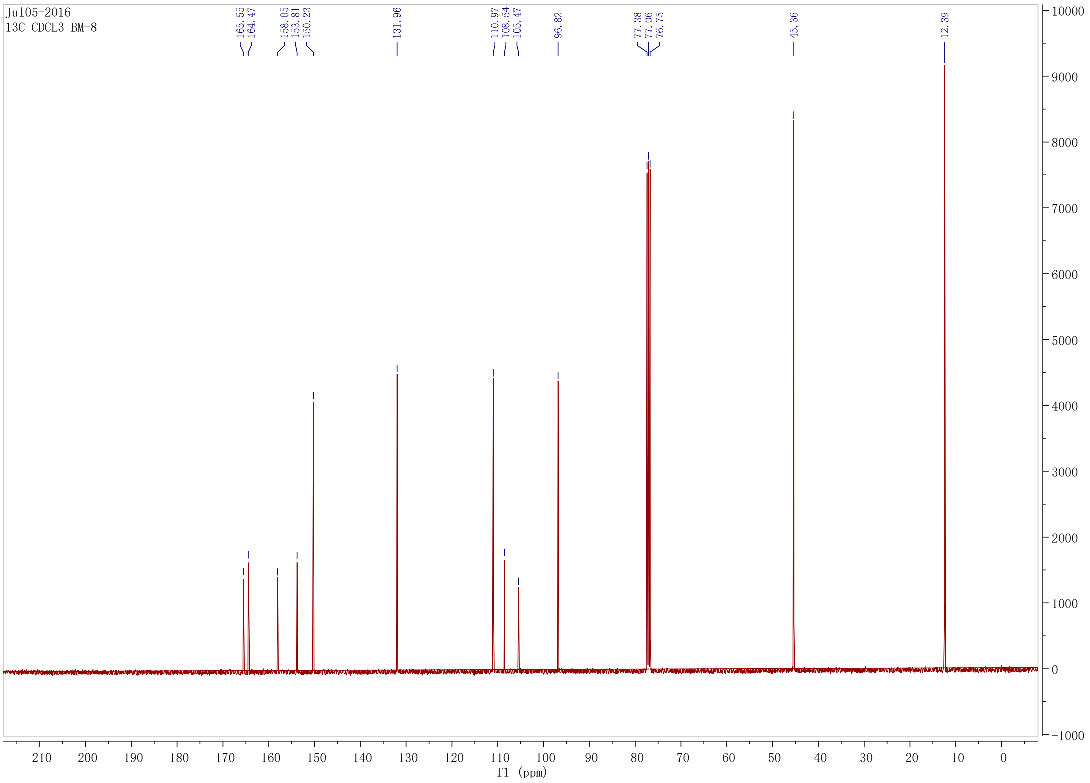

^{1}H NMR and ^{13}C NMR spectrums of **3a**

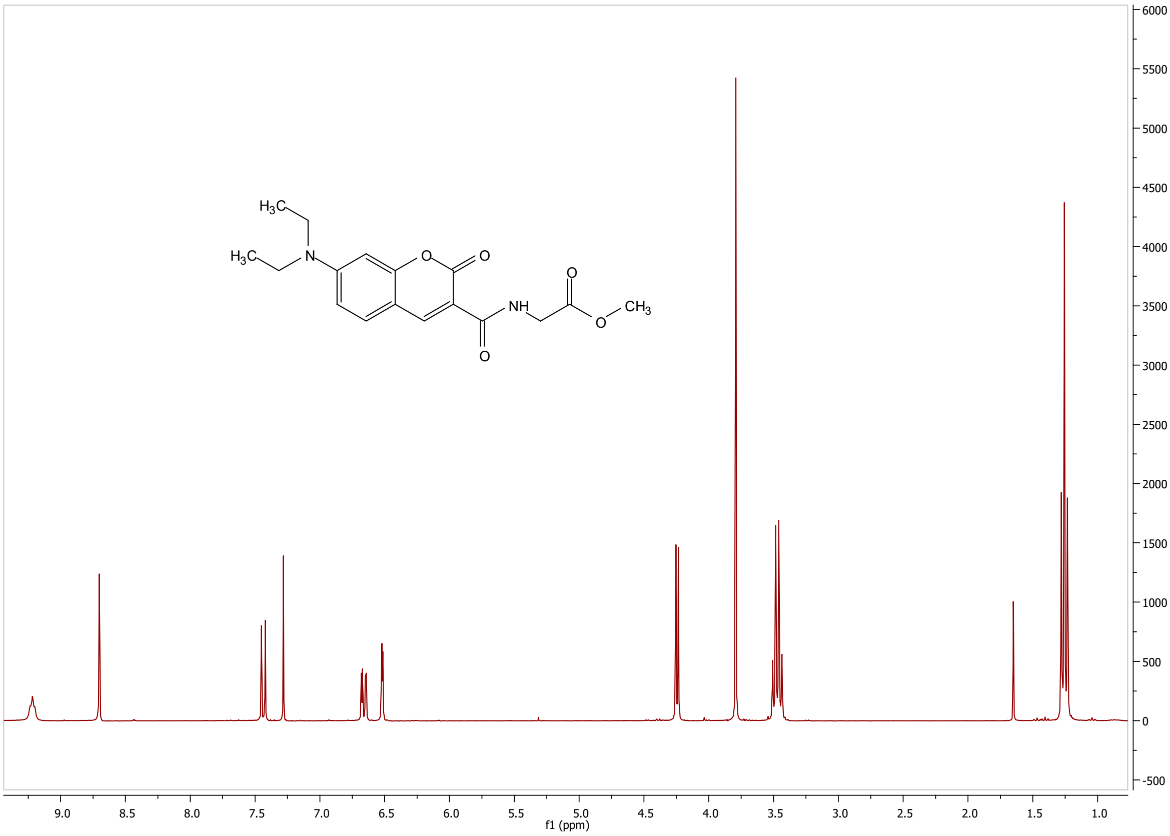

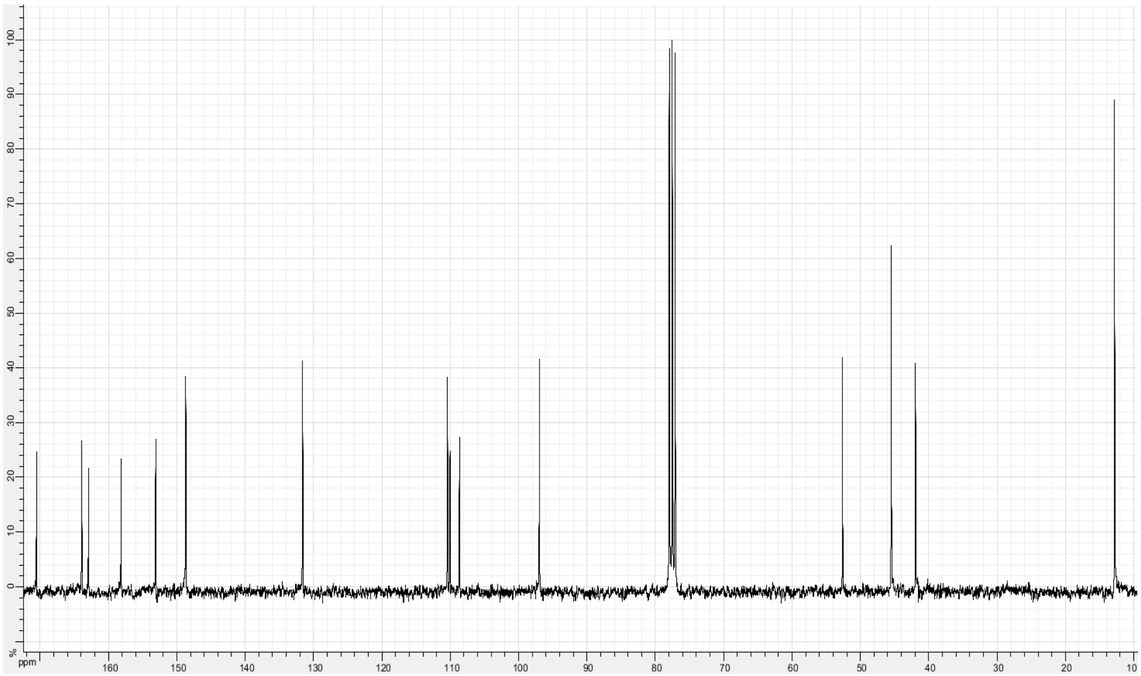

^{1}H NMR and ^{13}C NMR spectrums of **3b**

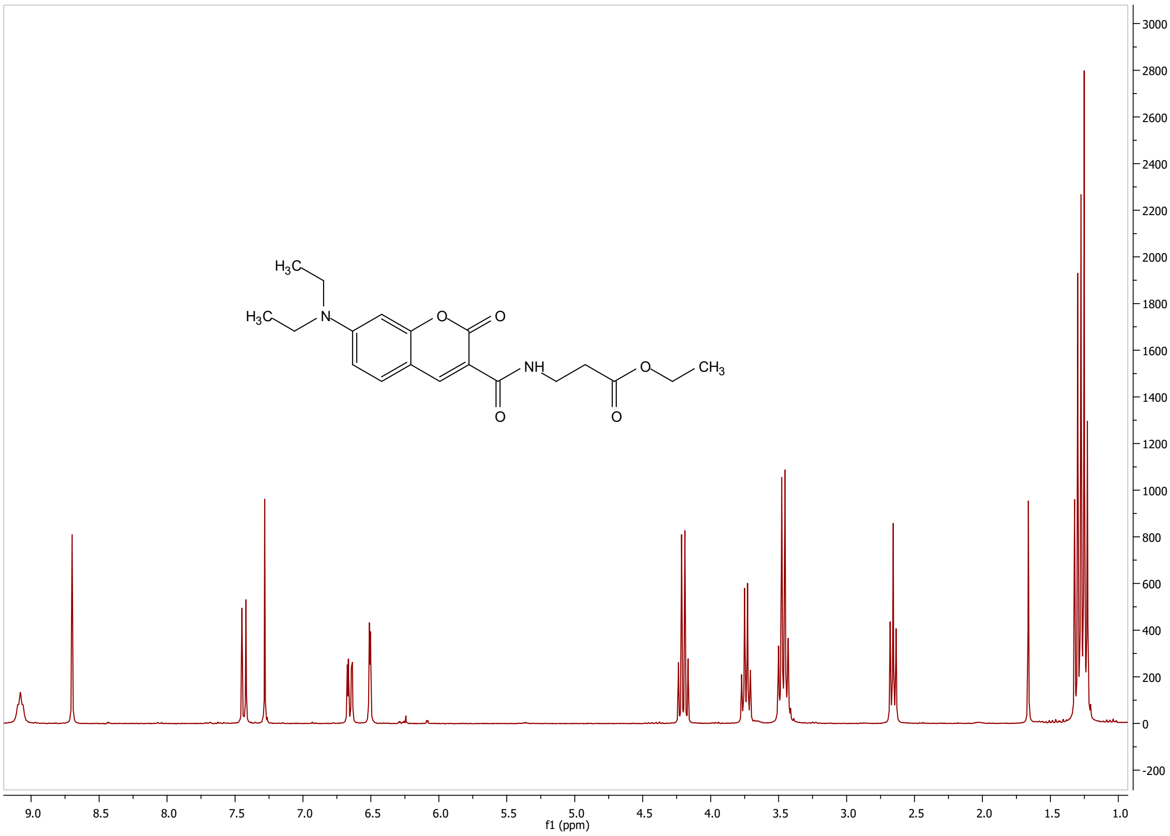

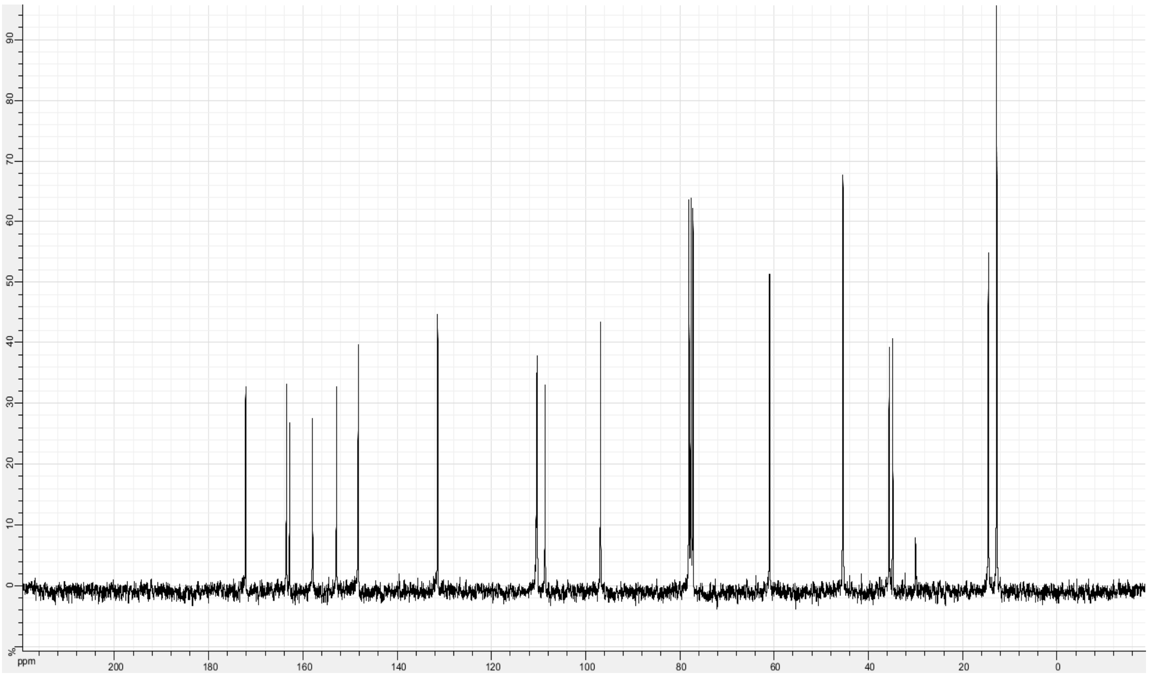

^{1}H NMR and ^{13}C NMR spectrums of **3c**

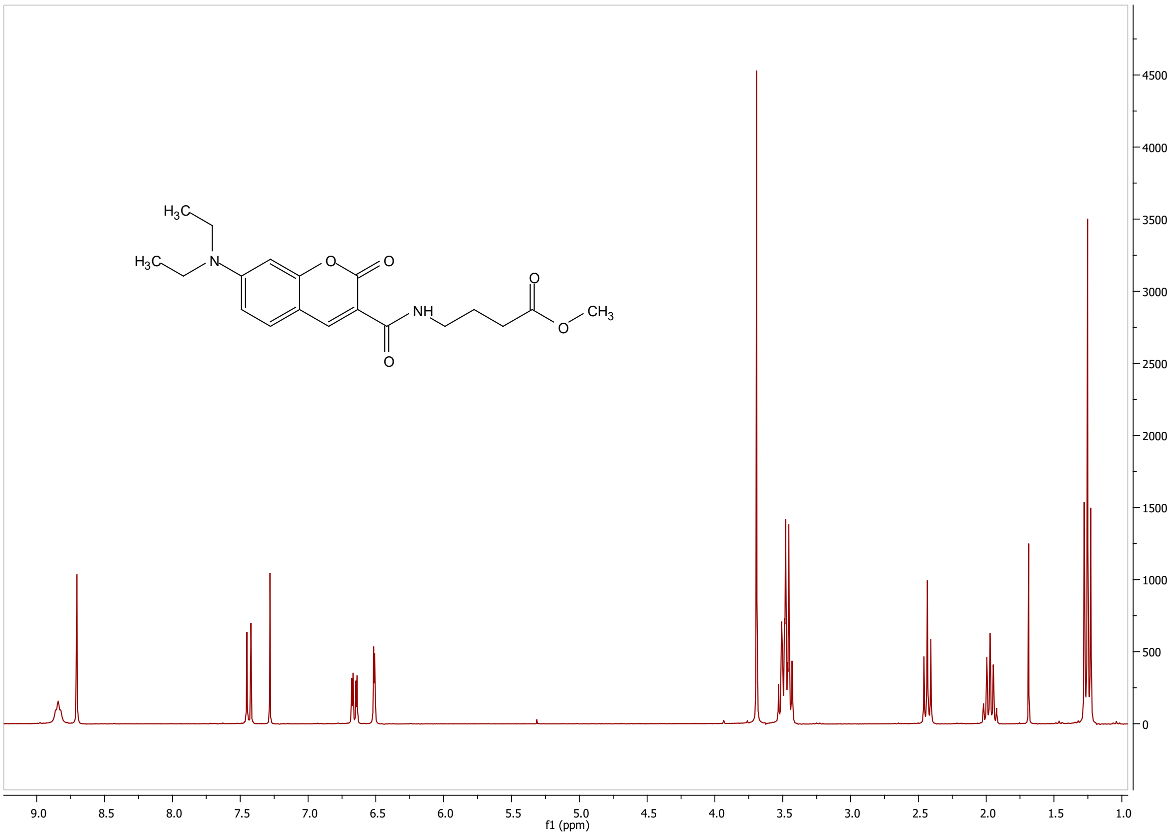

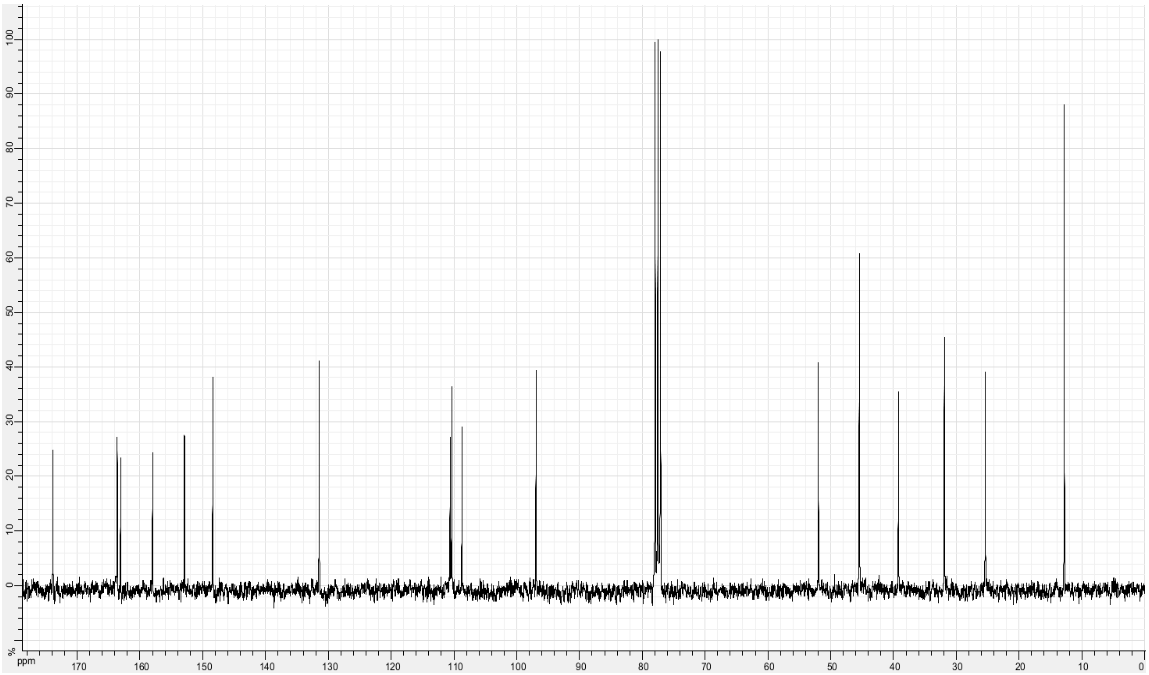

^{1}H NMR and ^{13}C NMR spectrums of **4a**

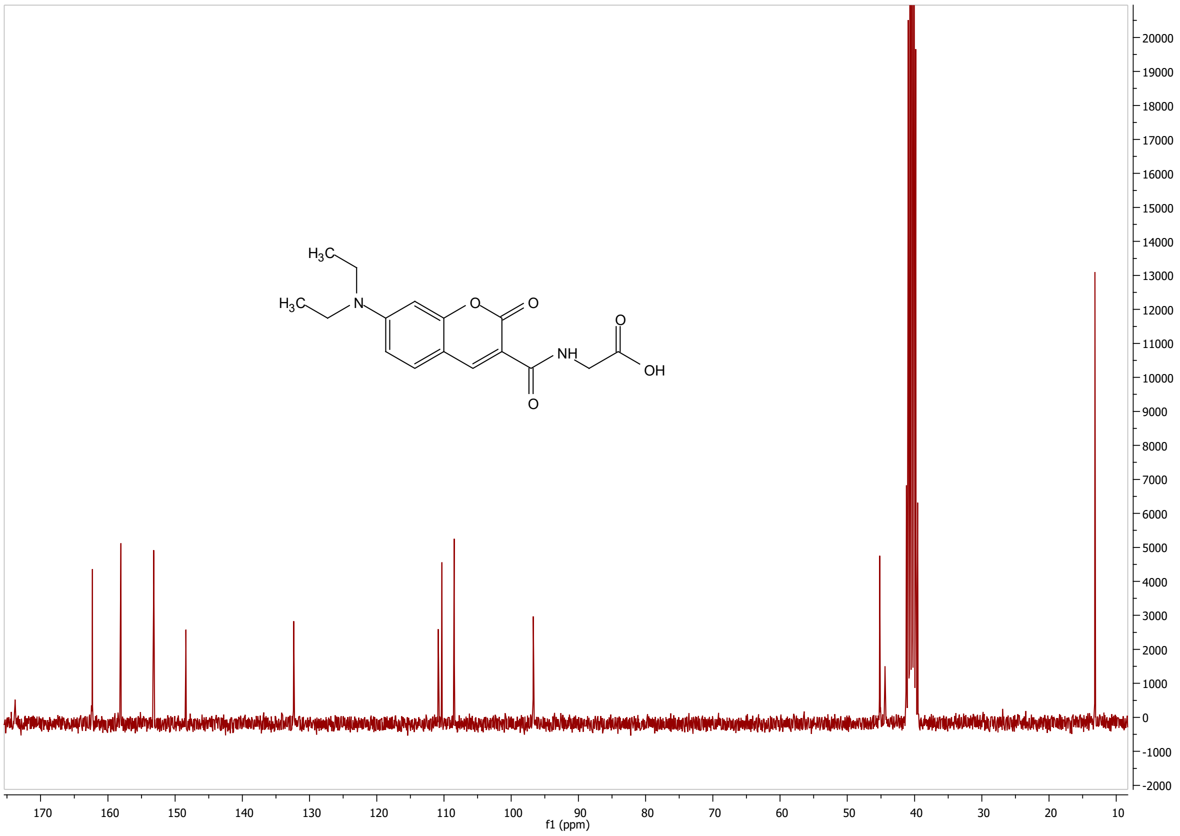

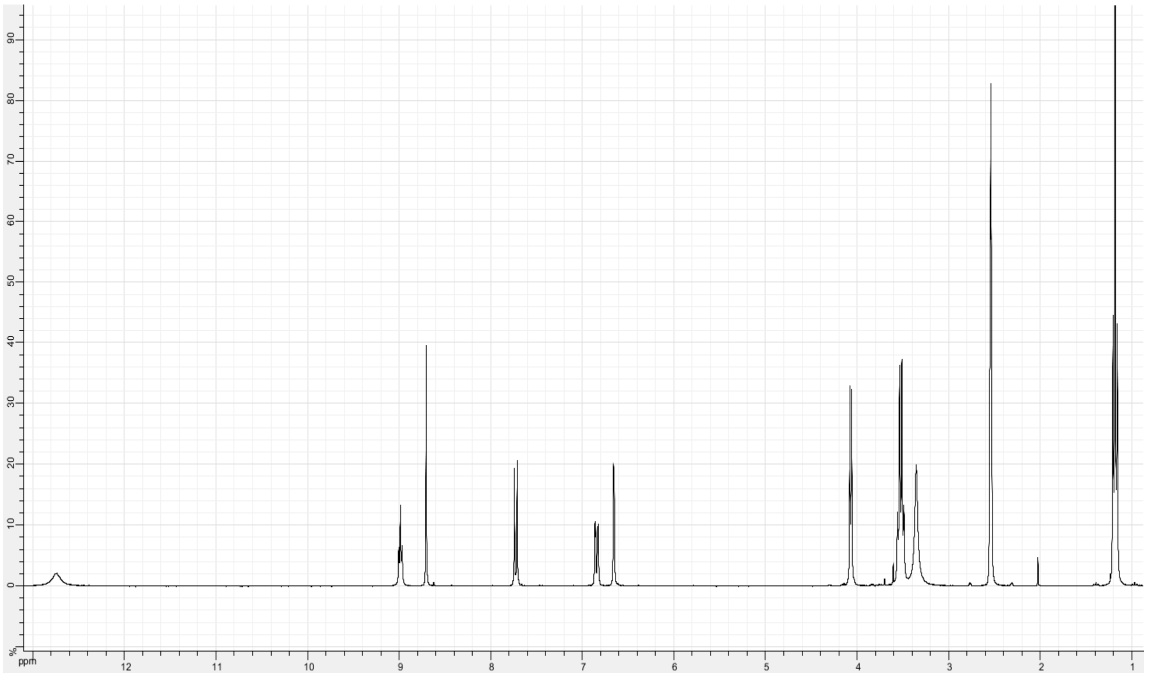

^{1}H NMR and ^{13}C NMR spectrums of **4b**

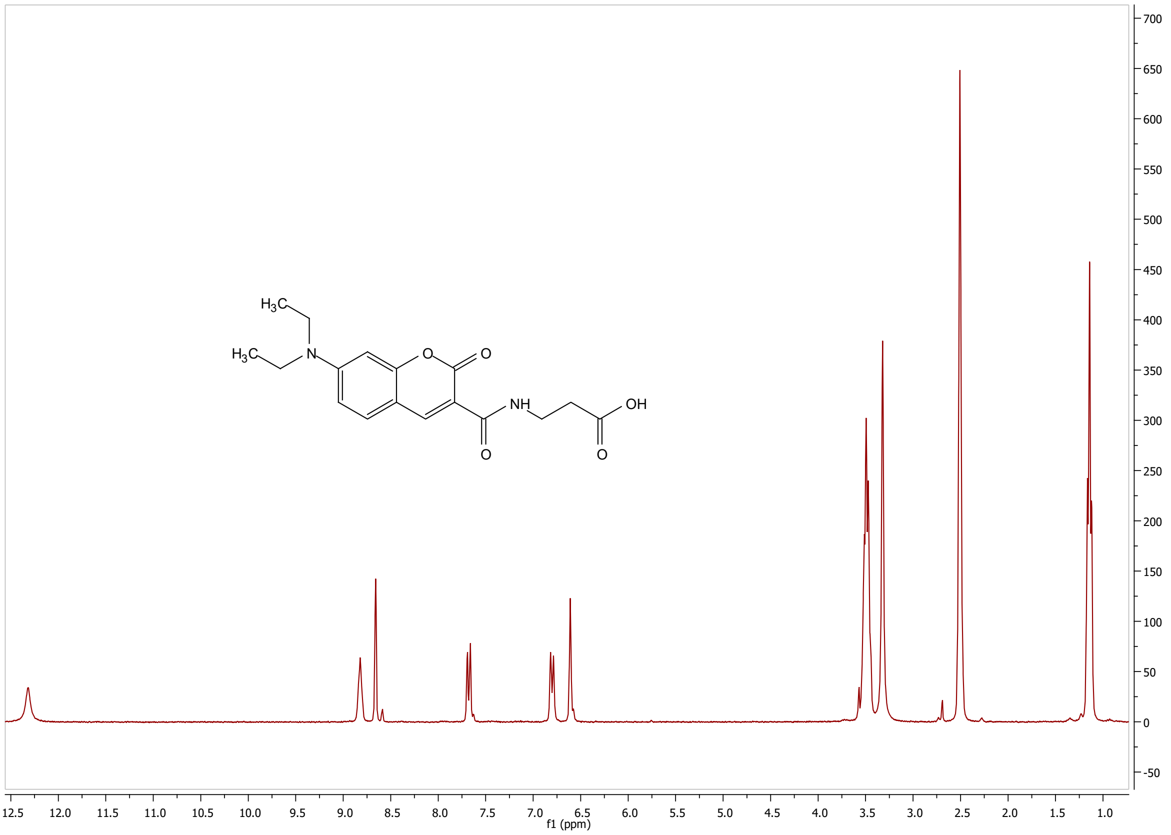

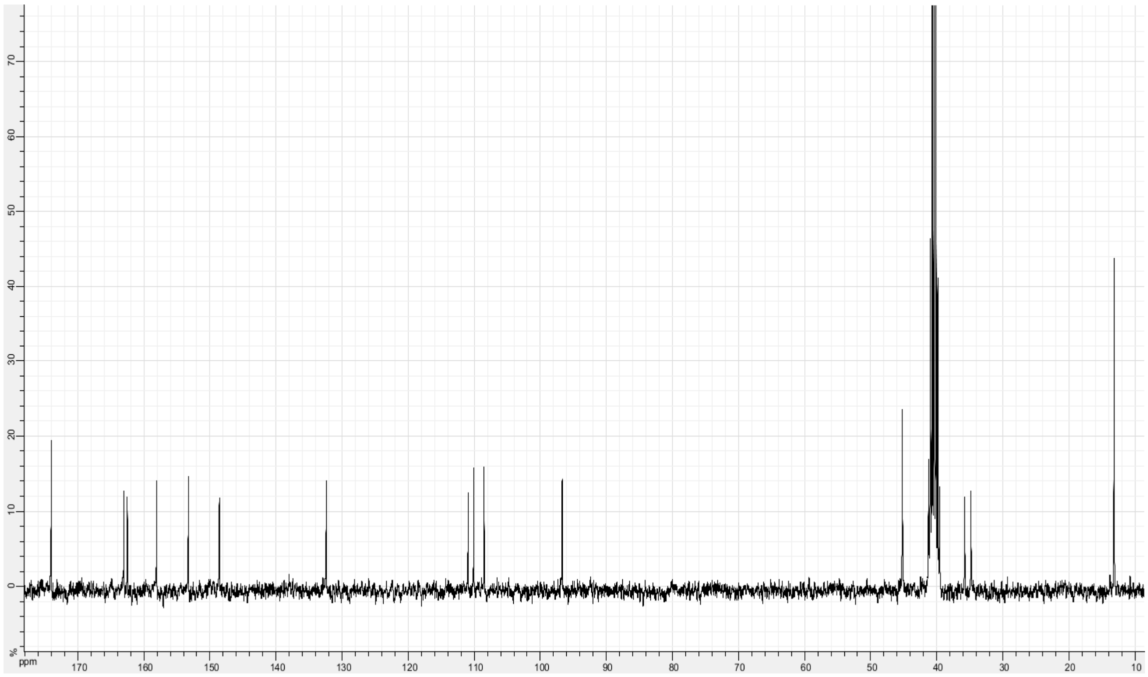

^{1}H NMR and ^{13}C NMR spectrums of **4c**

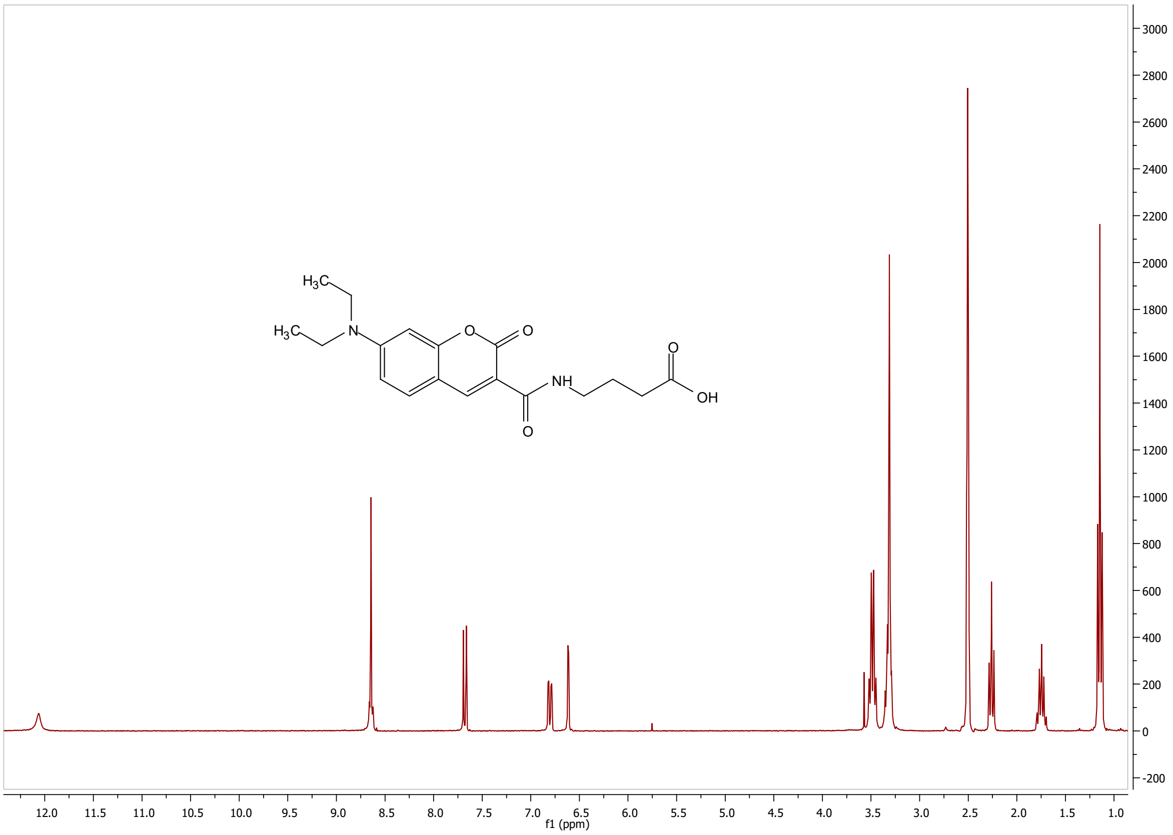

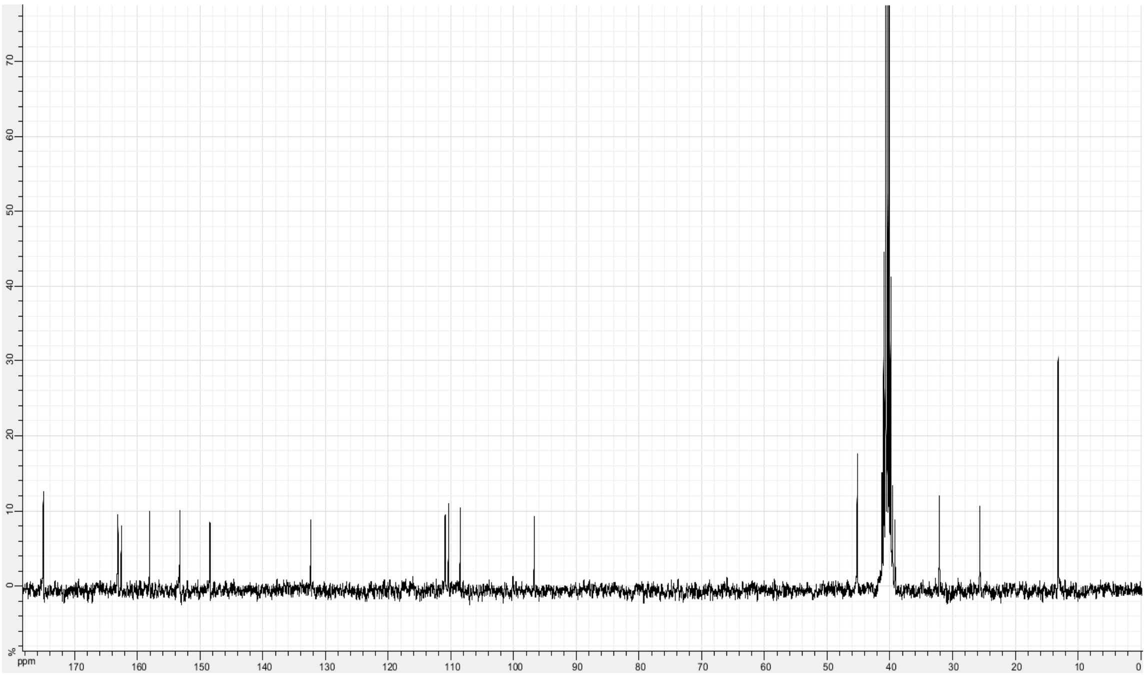

^{1}H NMR and ^{13}C NMR spectrums of **6**

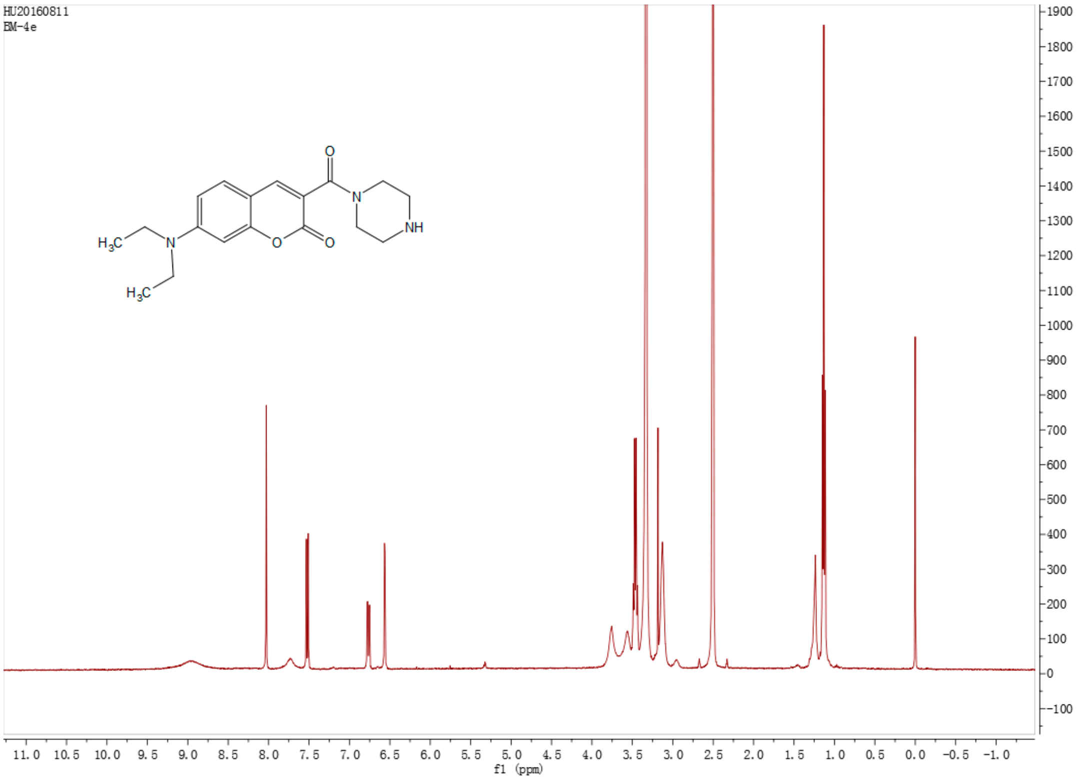

^{1}H NMR and ^{13}C NMR spectrums of **8a**

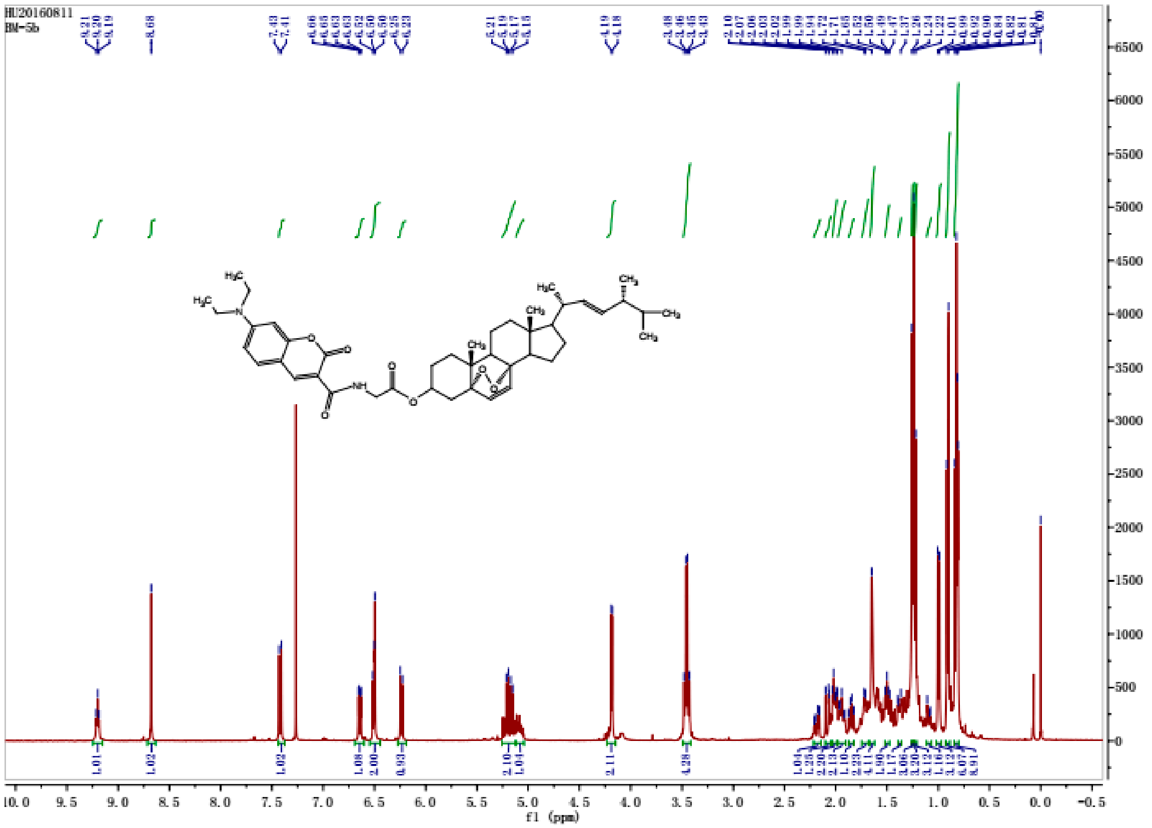

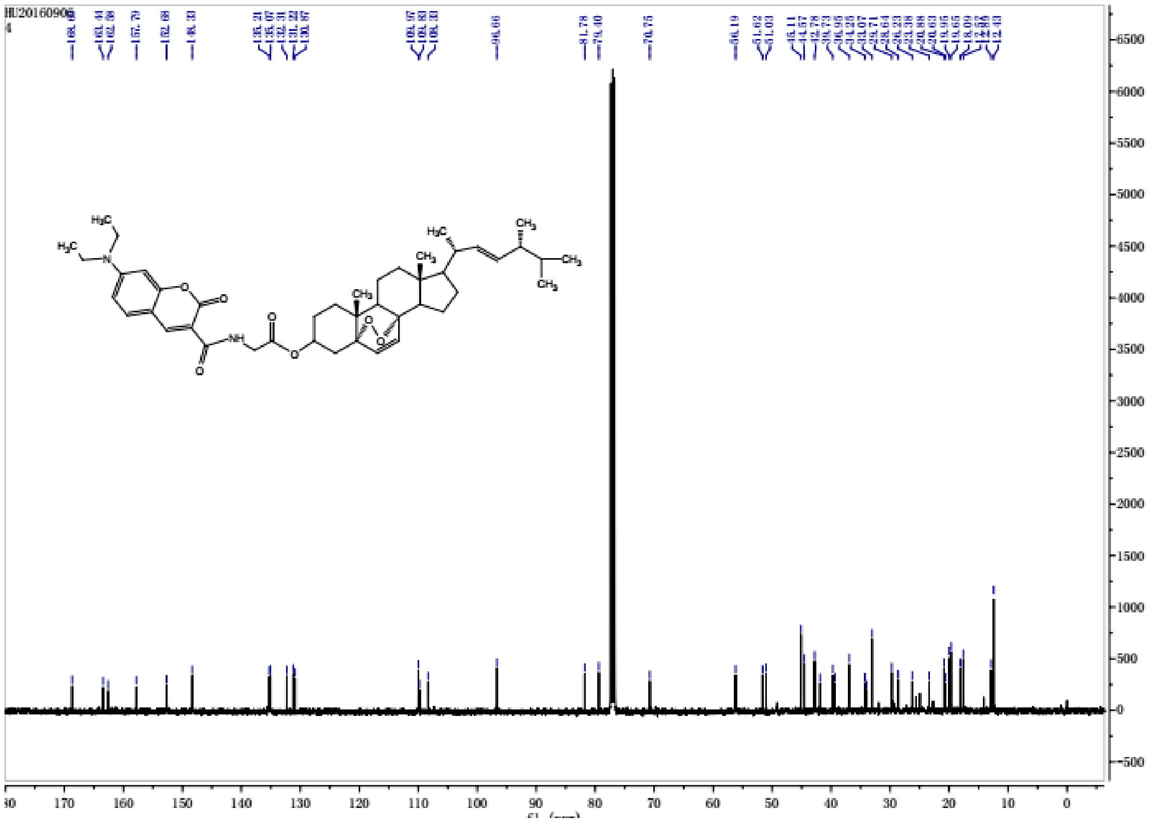

^{1}H NMR and ^{13}C NMR spectrums of **8b**

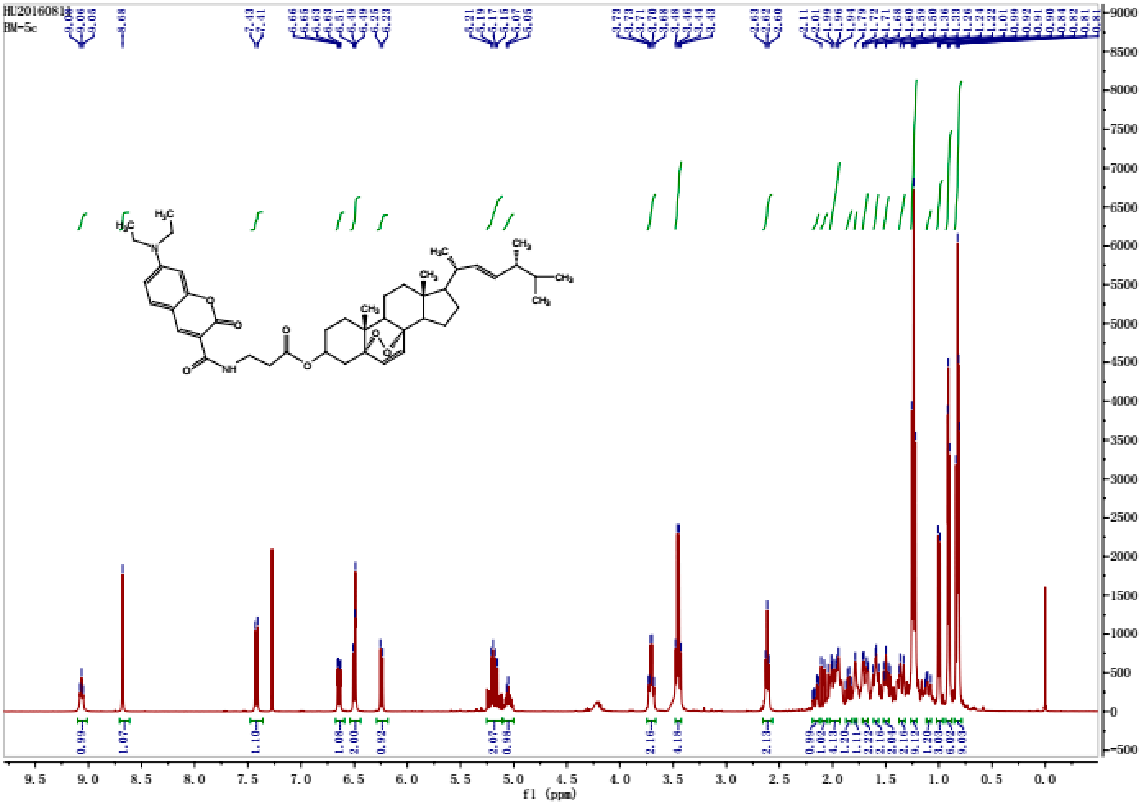

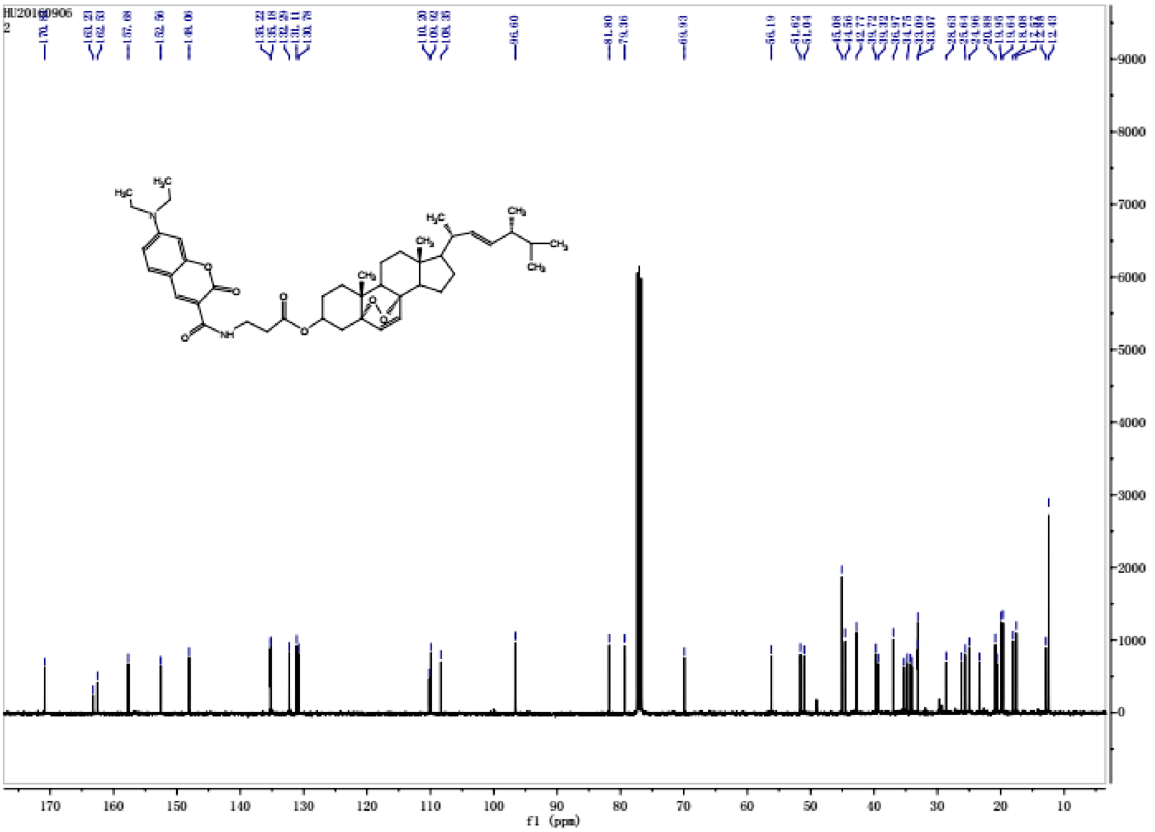

^{1}H NMR and ^{13}C NMR spectrums of **8c**

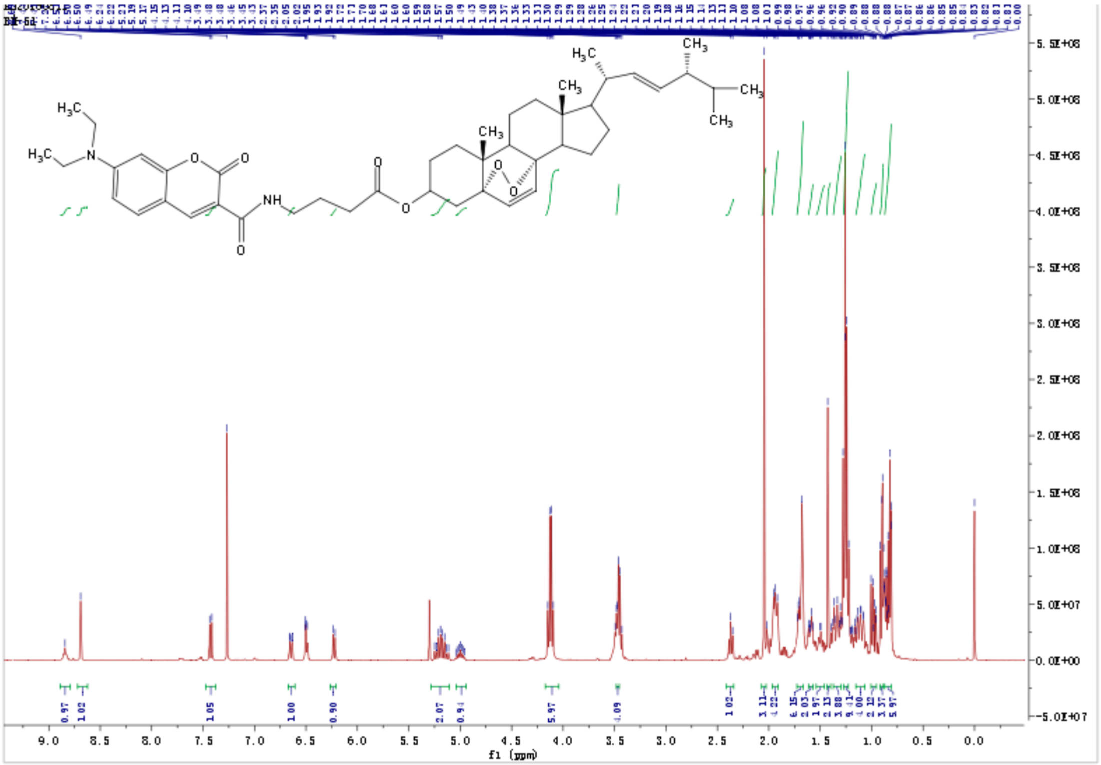

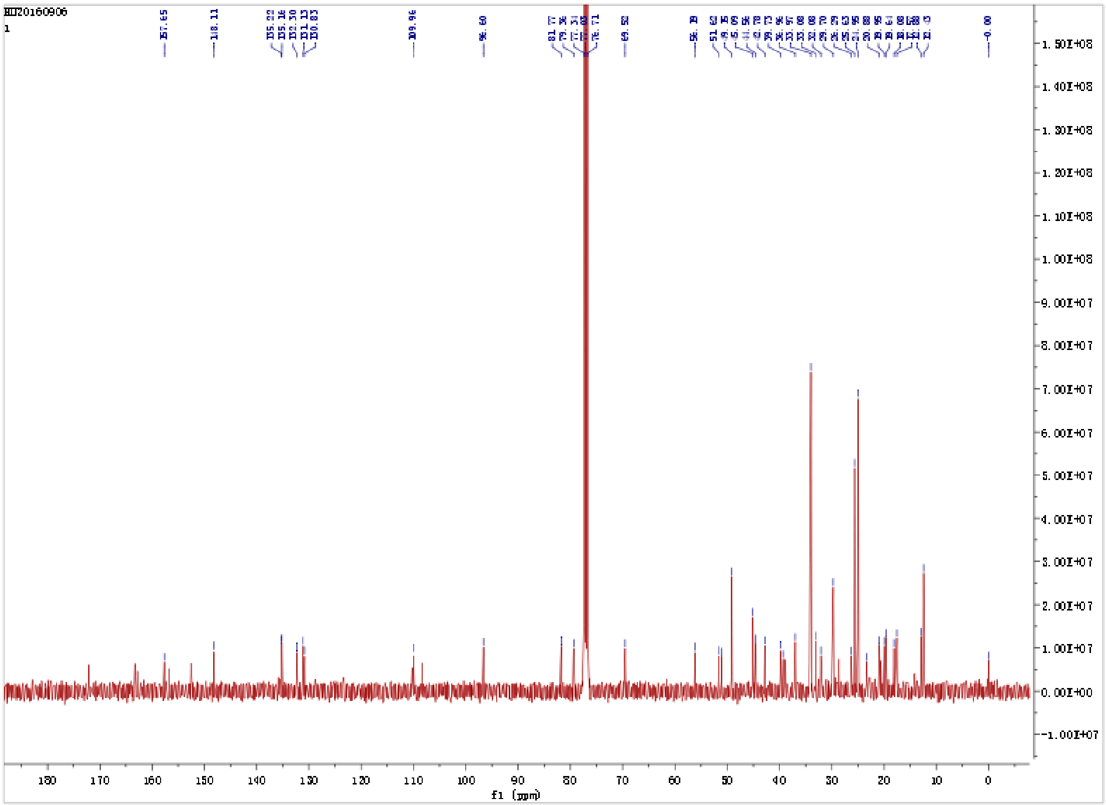

^{1}H NMR and ^{13}C NMR spectrums of **8d**

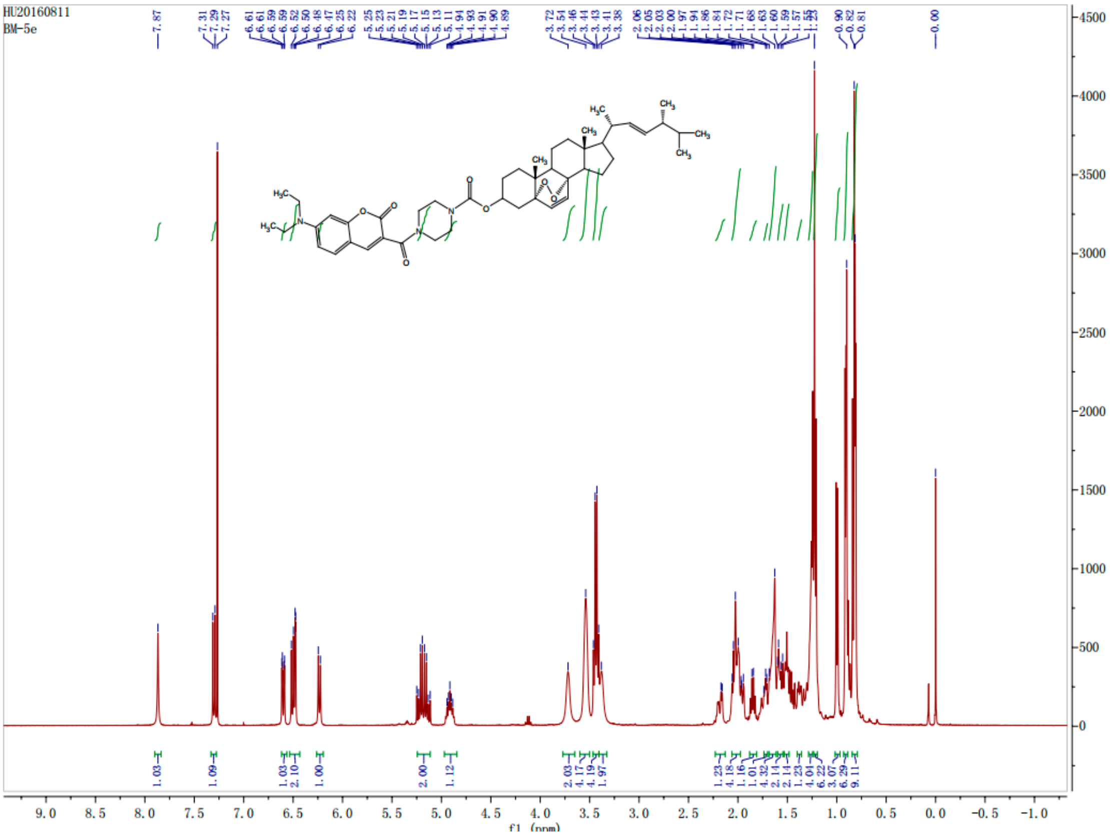

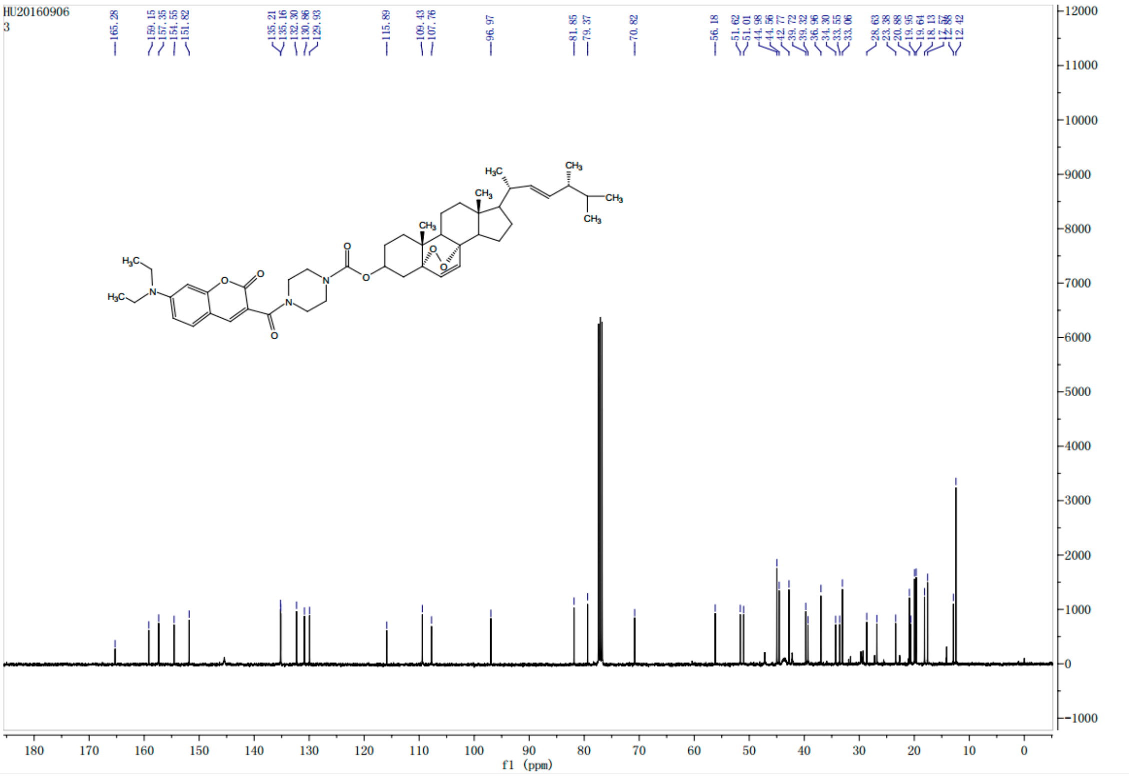

Supplement: Supplementary file 1 [file molecules-24-03307-s001.pdf]
